# Proteomic Analysis of Bladder Cancer Indicates Prx-I as a Key Molecule in BI-TK/GCV Treatment System

**DOI:** 10.1371/journal.pone.0098764

**Published:** 2014-06-06

**Authors:** Li Jiang, Xiao Xiao, Jin Ren, YongYong Tang, HongQing Weng, Qi Yang, MingJun Wu, Wei Tang

**Affiliations:** 1 Department of Urology, The First Affiliated Hospital of Chongqing Medical University, Chongqing, China; 2 Institute of Life Science, Chongqing Medical University, Chongqing, China; Louisiana State University Health Sciences center, United States of America

## Abstract

In order to understand the molecular mechanisms of Bifidobacterium infantis thymidine kinase/nucleoside analogue ganciclovir (BI-TK/GCV) treatment system which was proven to exhibit sustainable anti-tumor growth activity and induce apoptosis in bladder cancer, a proteomic approach of isobaric tags for relative and absolute quantification (iTRAQ), followed by liquid chromatography-tandem mass spectrometry (LC-MS/MS) was used. 192 down-regulated and 210 up-regulated proteins were identified after treatment with BI-TK/GCV system in Sprague-Dawley (SD) rats. Western blot analysis and immunohistochemistry analysis confirmed that Peroxiredoxin-I (Prx-I) was significantly down-regulated in bladder cancer after treatment. Prx-I silencing by transfection of Prx-I shRNA significantly suppressed growth, promoted apoptosis and regulated the cell cycle in T24 cells and reduced the phospho-NF-κB p50 and p65 protein expression which revealed the links between Prx-I and NF-κB pathway implied by Ingenuity pathway analysis (IPA). These findings yield new insights into the therapy of bladder cancer, revealing Prx-I as a new therapeutic target and indicating BI-TK/GCV system as a prospective therapy by down-regulation of Prx-I through NF-κB signaling pathway.

## Introduction

Bladder cancer is the most common urological cancer in Asia and its clinical management is extremely expensive [1]. In 2014, about 74,690 new cases of bladder cancer are expected to be diagnosed, and about 15,580 of them will die [2]. Bladder cancer can be divided into two major clinical and pathological subtypes: superficial non-muscle-invasive type and advanced muscle-invasive type [3]. In general, superficial bladder cancer is treated with endoscopic resection with favorable prognosis, but it sometimes reoccurs with grade progression [4]. The management of advanced bladder cancer is a major challenge, and these patients have a less favorable prognosis with a very low 5-year survival rate; therefore, more aggressive therapeutic options are necessary such as radical cystectomy and urinary diversion [5]. Further, bladder cancer especially the advanced type reoccurs in a substantial number of patients, and even results in death, and therefore, preferably less aggressive approaches should be developed to combat the disease.

Herpes simplex virus thymidine kinase (HSV-TK)–mediated suicide gene therapy as a widely accepted strategy for bladder cancer can convert the nontoxic nucleoside analog ganciclovir (GCV) into a toxic triphosphorylated form, which subsequently causes the death of rapidly dividing cells [6]. We previously resorted to *Bifidobacterium infantis* (BI) which is a tumor-targeting bacterium, because it selectively localizes and proliferates within the hypoxic regions of tumors as a non-pathogenic and anaerobic bacterium [7], [8]. We found that the BI and TK/GCV (BI-TK/GCV) system exhibited a sustainable anti-tumor growth activity in the rodent bladder cancer model in vivo, which involved both extrinsic and intrinsic apoptosis pathways [9].

In an effort to understand the underlying molecular mechanisms and identify the potential target protein molecule of this safe and effective treatment system, we resorted to mass spectrometry (MS)-based isobaric tags for relative and absolute quantification (iTRAQ) to obtain comprehensive differential protein profiles. Furthermore, we investigated the molecular pathway of Peroxiredoxin-I (Prx-I), one of the identified down-regulated proteins in bladder cancer identified by iTRAQ after treatment with BI-TK/GCV.

## Materials and Methods

### Materials

The BI-TK/GCV treatment system was constructed successfully by our research group (Chongqing, China) [9].

### Experimental animals

Seventy female Sprague–Dawley rats (6–8 weeks old, weighing 180–200 g) were purchased from Chongqing National Biological Industry Base of Experimental Animal Center (China) and housed under specific pathogen-free condition at 23–27°C and humidity 55–65% with 12-h light–dark cycles. All animal procedures were approved by the Animal Use and Care Committee of Chongqing Medical University. A rat bladder tumor model was built by perfusion of N-methyl-nitrosourea (MNU)(Sigma, USA). MNU was diluted into 20 g/l by a citric acid buffer solution. Each bladder was perfused with 0.1 ml once every 2 weeks, for a total of four perfusions.

### Studies in vivo

Sixty tumor-bearing Sprague-Dawley rats were randomly divided into four groups (each *n* = 15): a normal saline group, a BI group, a BI/PGEX-1 group, and a BI-TK group. After the bifidobacterium was concentrated, about 0.5 ml of the corresponding interventions was injected via the tail vein (bacterium count, 4.4×10 ^9^) once a week for 4 weeks. All groups received daily intraperitoneal injection of GCV (50 mg/kg) for 28 days. All the rats were sacrificed under anesthesia with sodium pentobarbital. Part of the bladder cancer tissues from the four groups were preserved in paraffin for immunohistochemical (IHC) analysis, and the rest of the tissues were kept at −80°C for further analysis.

### Protein sample preparation and iTRAQ labeling

The tissues were lysed in a lysis buffer (7 m urea, 1 mg/ml DNaseI, 1 mmNa_3_VO_4_, and 1 mm phenylmethane sulfonylfl uoride, PMSF) and centrifugated at 6000 g and 4°C for 30 min. The supernatant was collected and the total protein content was measured using 2D Quantification Kit (Amersham Biosciences, Uppsala, Sweden). Each sample was digested with 20 µl of 0.1 µg/µl trypsin solution (Promega, Madison, USA) at 37°C overnight and then labeled with the iTRAQ tags as follows: (i) normal saline group, 114 tags; (ii) BI-TK group, 115 tags; (iii) BI/PGEX-1 group, 116 tags; (iv) BI group, 117 tags. The labeled samples were pooled prior to further analysis.

### Strong cation exchange (SCX) chromatography

To reduce the sample complexity for liquid chromatography (LC)-MS/MS analysis, the pooled samples were diluted 10-fold with an SCX buffer A (10 mm KH_2_PO_4_ in 25% acetonitrile at pH 3.0) and utilized to a 2.1×200 mm polysulfoethyl A SCX column (PolyLC; Columbia, USA). The column was eluted with a gradient of 0–25% SCX buffer B (10 mM KH_2_PO_4_ at pH 3.0 in 25% acetonitrile containing 350 mM KCl) over 30 min, followed by a gradient of 25–100% SCX buffer B over 40 min. These SCX fractions were lyophilized in a vacuum concentrator and subjected to C-18 clean-up using extraction column (100 mg capacity, Supelco; Sigma-Aldrich, St. Louis, USA).

### Electrospray ion-quadrupole time-of-flight MS (ESI-Q-TOF-MS) Analysis

MS was performed using a nano-LC coupled online to a QStar Elite mass spectrometer (Applied Biosystems). The LC eluent was directed to an ESI source for Q-TOF-MS analysis. The mass spectrometer was set to perform information dependent acquisition (IDA) in the positive ion mode, with a selected mass range of 300–2,000 m/z. Peptides with +2 to +4 charge states were selected for tandem mass spectrometry, and the time of summation of MS/MS events was set to 3 s. Relative quantification of proteins, in case of iTRAQ, was performed on the MS/MS scans and was the ratio of the areas under the peaks at 113, 114, 115, and 116 Da, which were the masses of the tags that correspond to the iTRAQ reagents.

### Proteomic analysis

The bioinformatic processes and molecular function of the identified proteins in the BI-TK group after treatment were classified by the PANTHER classification system (www.pantherdb.org). The pathways of differentially expressed proteins identified by iTRAQ were analyzed by the Ingenuity pathway analysis (IPA) program (http://www.ingenuity.com). On the software Ingenuity, the data were used to extract interactive networks among the proteins in the International Protein Index (IPI) database. A network with a score higher than 2 is usually considered valid.

### Validation by Western blot and IHC

Western blotting and IHC were used to confirm the expression of Prx-I. T The protein samples (about 20 mg) were separated using SDS–PAGE. After SDS–PAGE electrophoresis, proteins were transferred to PVDF membranes. Subsequently, the lysates were incubated with a primary anti-Prx-I rabbit monoclonal antibody (1∶1500) (Abcam, USA). The immunoreactive signals were detected by enhanced chemiluminescence kit (Amersham Biosciences, Sweden). The procedures were conducted according to the manufacturer's instructions. Bladder cancer tissues from four groups were incubated overnight with primary antibodies. The tissues were incubated with secondary antibodies for 2 h. The cell nuclei were counterstained with hematoxylin. The proportion of positively stained tumor cells was determined by Image-Pro Plus (IPP) 6.0 and was graded as follows: 0, negative; 1, <10%; 2, 10–50%; 3, >50%. The immunostaining intensity was scored as follows: 0, absent; 1, light yellow; 2, yellowish brown; 3, brown. The protein in bladder cancer tissues was evaluated using the staining index (SI): *SI*  =  proportion × intensity of positive tumor cells.

### Prx-1 Knockdown by shRNA

T24 cells were subjected to Prx1 knockdown. Short hairpin RNA (shRNA) was exprssed with GV102 system (GeneChem, China). The three pairs of sense and antisense sequences of oligonucleotides targeting human Prx1(GeneBank_ID: NM_002574) were as follows: PRDX1-RNAi (sh-1) sense strand 5′-GATCCCGCTTTCAGTGATAGGGCAGAACTCGAGTTCTGCCCTATCACTGAAAGCTTTTTGGAT-3′, PRDX1-RNAi (sh-1) antisense strand 5′-AGCTATCCAAAAAGCTTTCAGTGATAGGGCAGAACTCGAGTTCTGCCCTATCACTGAAAGCGG-3′; PRDX1-RNAi (sh-2) sense strand 5′-GATCCCGATGAGACTTTGAGACTAGTTCTCGAGAACTAGTCTCAAAGTCTCATCTTTTTGGAT-3′, PRDX1-RNAi (sh-2) antisense strand 5′-AGCTATCCAAAAAGATGAGACTTTGAGACTAGTTCTCGAGAACTAGTCTCAAAGTCTCATCGG-3′; PRDX1-RNAi(sh-3) sense strand 5′-GATCCCCCATGAACATTCCTTTGGTATCTCGAGATACCAAAGGAATGTTCATGGTTTTTGGAT-3′, PRDX1-RNAi(sh-3) antisense strand 5′-AGCTATCCAAAAACCATGAACATTCCTTTGGTATCTCGAGATACCAAAGGAATGTTCATGGGG-3′.A scrambled sequence of the Prx-I target was used as a negative control (con sh). T24 cells were transiently transfected with the Prx1-shRNA expression vectors by using Lipofectamine 2000 (Invitrogen, USA). Non-infected T24 cells (parental) and Prx-I control shRNA infected T24 cells (con sh) were also used. The highest efficiency of knockdown by shRNA vectors in T24 cells was determined by quantitative real-time polymerase chain reaction (qRT-PCR). The primer sequences for Prx-I were 5′-AGCCTGTCTGACTACAAAG-3′ (forward) and 5′-TCTGCCCTATCACTGAAAG-3′ (reverse), which yielded a 104 bp product. The primer sequences for the internal control GAPDH were 5′-CACCCACTCCTCCACCTTTG-3′ (forward) and 5′-CCACCACCCTGTTGCTGTAG-3′ (reverse), which yielded a 110 bp product. The expression value of Prx-I compared with that of GAPDH was calculated as 2-ΔΔCt. All reactions were conducted in triplicate. Then the expressions of Prx-I in T24 cells knocked down by shRNAs were measured by Western blotting.

### Cell proliferation assay

After transfection with Prx-I shRNA for 24 h, the T24 cells were seeded into 96-well culture plates at a density of 4×10^3^ cells in a final volume of 100 µl/well, and the untransfected cells were used as a control. The cell proliferation rate was calculated at different time points (24, 48 and 72 h) using Cell Counting Kit-8 (CCK-8) (Sigma, USA). Experiments were performed according the manufacturer's protocol. The absorbance at 450/630 nm was measured with a Thermo spectrophotometer (Waltham, USA). The average absorbance from six wells per group was calculated.

### Flow cytometric analysis

After transfection for 48 h, the cells were trypsinized and centrifuged at 1500 rpm for 5 min. The cells were harvested and washed with PBS twice. After stained with 50 µg/ml Annexin V-fluorescein isothiocyanate (FITC) (BD Biosciences, USA) and 20 µl of 500 µg/ml propidium iodide (PI) (Sigma, USA) for apoptosis detection, the cells were incubated in dark at room temperature for 15 min and subjected to flow cytometry analysis (FACS). Then the cells were collected, washed with PBS, fixed with 75% ethanol at −20°C overnight. The fixed cells were washed with cold PBS twice, added 500 µL DNA staining solution (including 200 µg/mL RNase A and 20 µg/mL propidium iodide staining solution) and incubated for 30 minutes. Finally, the cells were subjected to cell cycle analysis by FACS. The data were analyzed and evaluated on the program ModFit (Topsham, USA).

### Effect of Prx-I on phospho-NF-κB p50 and p65

The link between Prx-I and the NF-kappa-B (NF-κB) complex signaling had been implied in the protein pathway of IPA. Therefore, in order to explore whether the effect of Prx-I on apoptotic signaling proteins was attributable to NF-κB inhibition, we evaluated nuclear levels of phospho-NF-κB p50 and p65 (Abcam, USA) by Western blot.

### Statistical analysis

The data were expressed as mean ± standard deviation (SD) and compared using analysis of variance. The level of significant difference was defined as p<0.05. All analyses were performed on SPSS 18.0 (SPSS, Chicago, USA) for Windows.

## Results

### Quantification and identification of differentially expressed proteins by iTRAQ

**Figure 1:**
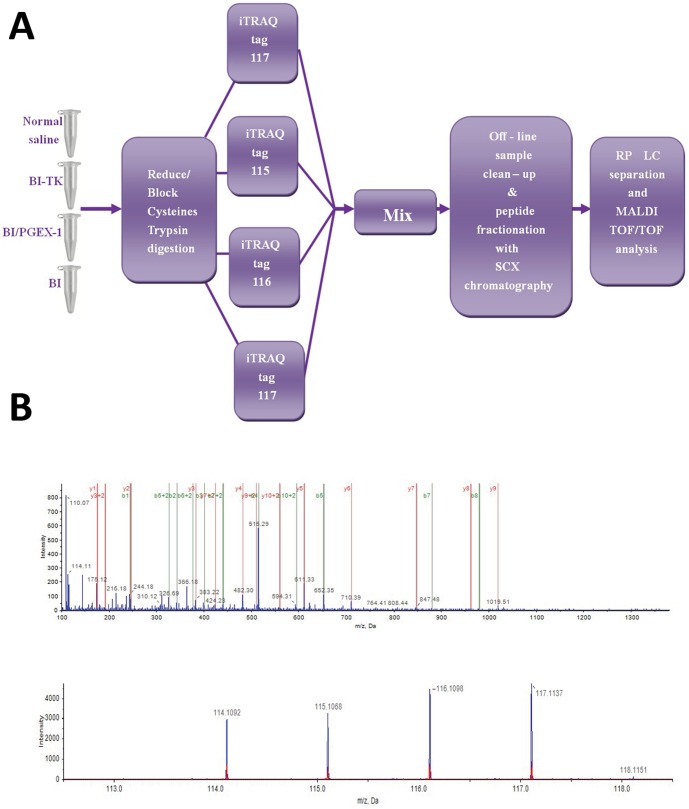
Experimental design of proteome analysis after treatment using iTRAQ labeling. A: Schematic diagram showing the workflow of iTRAQ. B: MS/MS spectrum showing the peptides of Prx-I (peptide sequence: VVGGDHVEVHAR). The 4 peak contours describe that the sample volumes are the same which guarantees the results are authentic and reliable.

A total of 2343 unique proteins were identified with 95% confidence by the ProteinPilot search algorithm against the IPI rat protein database v3.49. A strict cutoff value of a 1.3-fold change resulted in a final set of 402 differentially expressed proteins, including 192 down-regulated proteins and 210 up-regulated proteins in the BI-TK group after treatment. Strikingly, a novel molecule Prx- I drew our particular attention, with a 0.52-fold decrease in the BI-TK group versus the normal saline group. A schematic diagram of iTRAQ is shown in Figure 1A, and the MS/MS spectrum of Prx- I (peptide sequence: VVGDHVEVHAR) is shown in Figure 1B. The iTRAQ tags are as follows: (i) the normal saline group, 114 tags; (ii) the BI-TK group, 115 tags; (iii) the BI/PGEX-1 group, 116 tags; (iv) the BI group, 117 tags. The protein ID in IPI, name and main functions with abundance changes are epitomized in Table S1.

### Bioinformatic functional analysis of differentially expressed proteins after treatment with BI-TK/GCV

**Figure 2:**
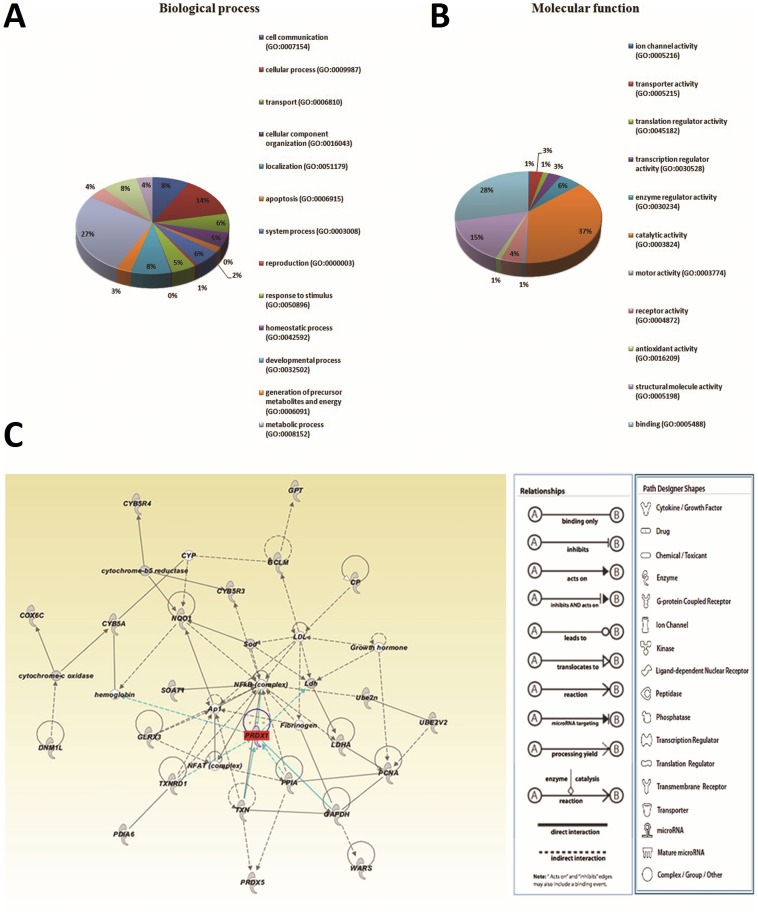
Bioinformatic analysis of differentially expressed proteins after treatment with BI-TK/GCV. PANTHER classification of proteins based on (A) Biological process and (B) molecular function. (C) Interplaying network of proteins with abundance change generated by Ingenuity pathway analysis (IPA). The network implied the connection of Prx-I and NF-κB complex.

To probe into their biological roles in the curative effect of BI-TK/GCV on bladder cancer, the differentially expressed proteins were categorized into various processes and function classes based on PANTHER classification system. In biological process analysis, the largest proportion of differentially expressed proteins was in metabolic process, followed by cellular process and cell communication process (Figure 2A). Notably, the proteins involved in catalytic activity, binding, structural molecule activity, enzyme regulator activity, and receptor activity were the top five molecular function categories (Figure 2B). Moreover, proteins involved in antioxidant activity accounted for 1.3%, respectively. Figure 2C depicts the primary pathways generated by IPA of the differentially expressed proteins. This network scored 36 and consisted of 37 proteins involved in apoptosis, oxidative stress, and metabolism. In particular, Prx-I, the markedly down-expressed protein after treatment, is directly linked to transcription factor NF-kappa-B (NF-κB) complex pathway in this network, indicating that Prx-I may play an important role in the apoptosis of bladder cancer by BI-TK/GCV system partly through the NF-κB signaling pathway.

### Validation of Prx-I by Western blot and IHC

**Figure 3:**
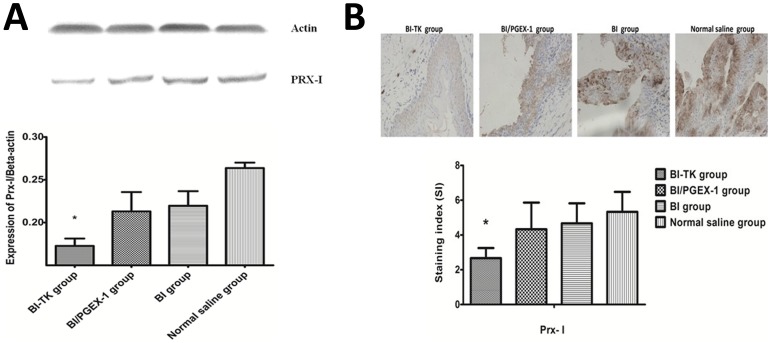
Validation of Prx-I by Western blot and immunohistochemical (IHC) analysis. A: Expressions of Prx-I in four groups by Western blot analysis. Beta-actin was used as a loading control. B: Representative images showing the immunoexpression of Prx-I in tumor tissues of four groups. Compared with the normal saline group, expression of Prx-I is down-regulated in the other three groups (especially in the BI-TK group), which is similar to the results obtained by iTRAQ. (Asterisk (*) indicates P<0.05 in BI-TK group versus normal saline group)

The differential expression levels of Prx- I identified by iTRAQ approach were validated by Western blot (Figure 3A). Compared with the normal saline group, expression of Prx-I is down-regulated in the other three groups (especially in the BI-TK group), which is similar to the results obtained by iTRAQ. Moreover, the Prx-I expression in the tumor tissues was verified by IHC analysis (Figure 3B). The Prx-I content in bladder cancer cells of BI-TK group was significantly lower than that in the other groups (p<0.05, Figure 3B), which is consistent with the results obtained by iTRAQ and Western blot.

### qPCR and Western blot for the interfering efficiency in T24 cells

**Figure 4:**
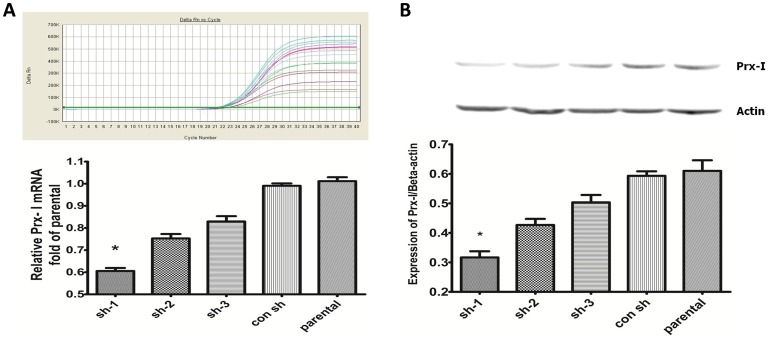
Suppression of Prx-I expression with the shRNA vectors in T24 cells. A: The expression of Prx-I mRNA was examined by qPCR. GAPDH served as an internal control. B: The Prx-I protein levels were analyzed by Western blot after transfection. Sh-1 treatment led to a significant reduction in Prx-I protein expression in T-24 cells. (Asterisk (*) indicates P<0.05 in sh-1 group versus parental group)

First, the Prx-I mRNA levels from four shRNA vectors transfected for 48 h in the T24 cell lines were measured by qPCR using Lipofectamine 2000. The Prx-I expression decreased by ∼40%, 26%, 18% and 1% in the sh-1, sh-2, sh-3 and con sh groups, respectively compared to the parental group (Figure 4A). To confirm this interfering efficiency, the protein expression levels of Prx-I in T24 cells after transfection were examined by Western blot. Results showed that Prx-I levels decreased significantly by ∼48%, 30%, 18% and 3% from baseline in the sh-1, sh-2, sh-3 and con sh groups respectively (Figure 4B), indicating that the highest interfering efficiency in T24 cells was in the sh-1 group.

### Prx-I knockdown inhibited T-24 cell growth

**Figure 5:**
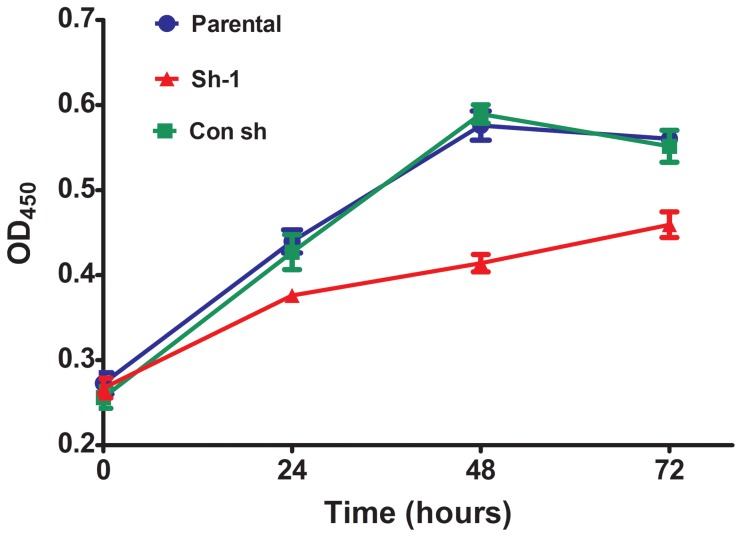
Prx-I knockdown inhibited the proliferation of T24 cells in vitro. The growth rates in Prx-I knockdown group was significantly reduced, compared with the parental and con sh groups, measured by CCK8 assay.

The effects of Prx-I shRNA transfection on T24 cell growth were investigated through CCK8 assays. A slight inhibition in growth was observed at 24 h after transfection. Furthermore, obvious inhibitory effects on cell proliferation were observed in Prx-I knockdown-cells at 48 and 72 h, compared with the parental and con sh groups (Figure 5, p<0.05), suggesting that the inhibition of Prx-I could suppress T24 cell growth in vitro.

### Effect of Prx-I shRNA transfection on the apoptosis and cell cycle of T-24 cell

**Figure 6:**
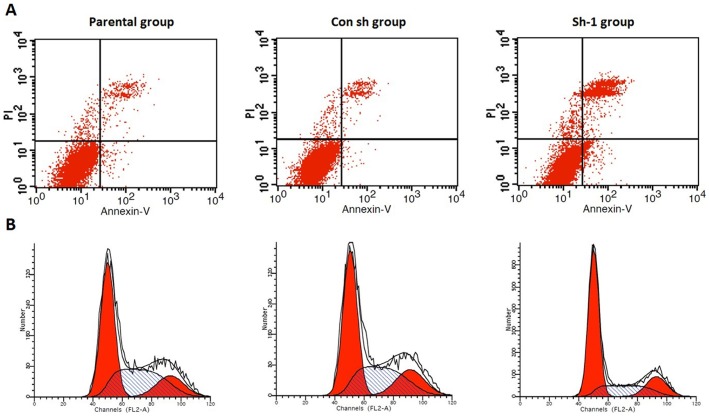
Effect of Prx-I-shRNA transfection on the apoptosis and cell cycle of T24 cells. A: Prx-I knockdown induced apoptosis in T24 cells. B: Representative pictures of FACS analysis showing Prx-I knockdown induced G0/G1 cell cycle arrest in T24 cells with a corresponding decrease in S-phase cells (P<0.05).

The effects of Prx-I knockdown on apoptosis and cell cycle of T24 cell were investigated. After 48 h of transfection, the apoptosis rate in the sh-1 group (21.99±1.10%) was significantly higher compared with the con shRNA group (4.51±0.73%) and parental group (4.96±0.46%) (P<0.05) (Figure 6A). Cell cycle analysis showed that G0/G1 phase ratio in the sh-1 group (61.13±.50%) was significantly higher compared with the con sh group (49.62±0.84%) and parental group (48.03±1.17%) (P<0.05) (Figure 6B).

### Effect of Prx-I on NF-κB pathway

**Figure 7:**
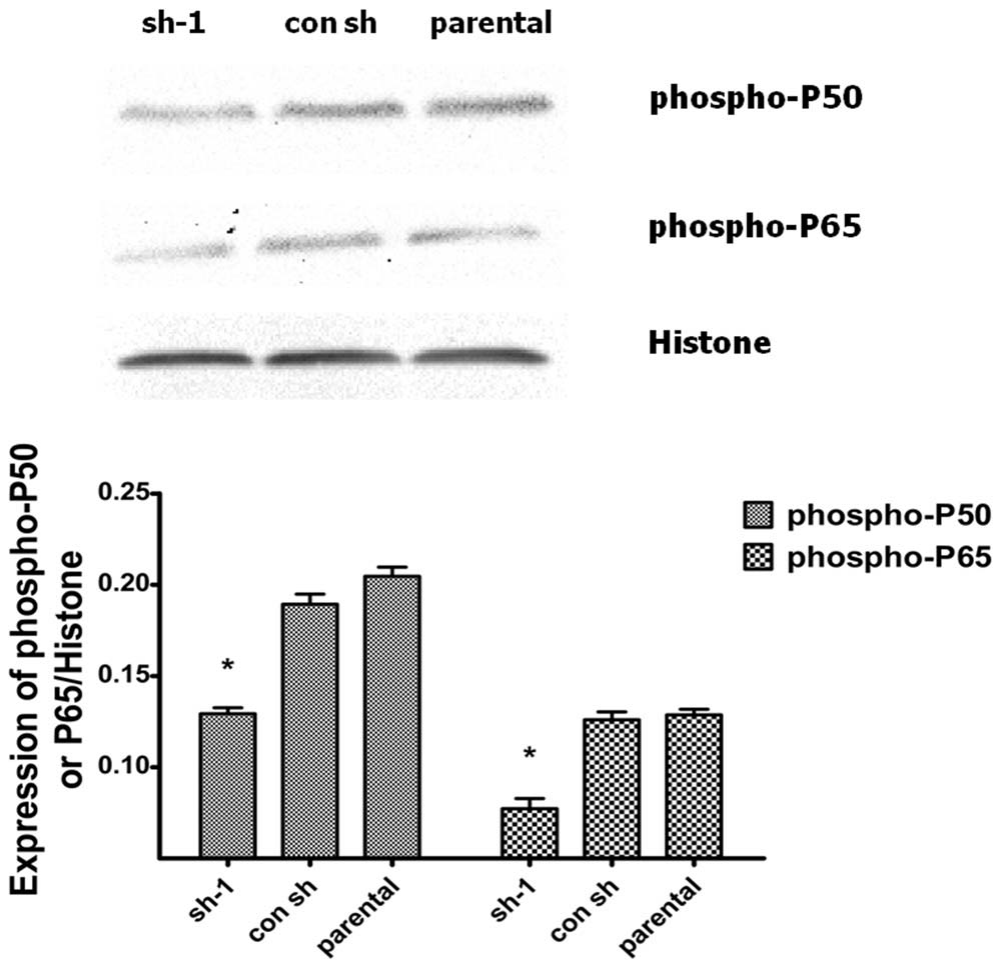
Effect of Prx-I-shRNA transfection on protein expression of phospho-NF-κB p50 and p65 in T24 cells by Western blots. A significant decrease in the protein expression of both phospho-NF-κB p50 and p65 in sh-1 group. (Asterisk (*)indicates P<0.05 in sh-1 group versus parental group)

As described above, Prx-I is directly linked to NF-κB complex, which has been implied in the protein pathway (Figure 2C). To explore whether the effects of Prx-I on apoptotic signaling proteins were attributable to NF-kB inhibition, the activated forms of phospho-NF-κB p50 and p65 were examined by Western blot after transfection with Prx-I shRNA in T24 cells. A significant decrease (P<0·05) in the protein expression of both phospho-NF-κB p50 and p65 was observed in sh-1 group (Figure 7), compared with the con sh and parental groups, indicating that Prx-I knockdown inhibited activation of NF-κB P50 and P65 in T24 cells.

## Discussion

In our previous study, BI-TK/GCV was constructed and proved effective in inhibiting the progressive growth of bladder tumor which was related to apoptosis in vivo [9], [10], indicating that BI-TK/GCV was a successful treatment system and might provide a novel strategy for treatment of advanced or metastatic bladder cancer in future.

In this study, a proteomic approach iTRAQ was used to identify differentially expressed proteins, aiming to reveal the molecular mechanisms and provide theoretical support for the effectiveness of BI-TK/GCV system. iTRAQ identified 402 differentially expressed proteins in bladder cancer tissues after treatment, including 192 downregulated proteins and 210 upregulated proteins. The targeting proteins with differential abundance (Table S1) played pivotal roles in a number of cellular pathways, including metabolism, apoptosis, antioxidant activity, cell cycle, proliferation, signal transduction and cell adhesion. Although the exact mechanism by which BI-TK/GCV reaches its intracellular targets is unclear, the targeting proteins of BI-TK/GCV are supposed to participate in the proliferation and apoptosis of bladder cancer cells, and provide novel targets for future therapy.

Prx-I stood out in our proteomic analysis because it had rarely been linked directly with bladder cancer although it has been shown to down-express in bladder cancer tissues after treatment with BI-TK/GCV. The mammalian peroxiredoxin (Prx) family which consists of six proteins is H_2_O_2_-scavenging enzymes present in procaryotic and eucaryotic cells [11]–[13]. Prx-I belongs to typical-2-Cys and is the most abundant and ubiquitously distributed isoform of PRDX [14], [15], which has been closely interrelated with cell proliferation and differentiation, intracellular redox signaling, and apoptosis [16]–[19]. Prx-I is highly expressed in solid organs and in tissues of some cancers [20]–, and is also positively associated with the recurrence and progression rates of bladder cancer [24], [25]. Similarly, Prx-I over-expression is associated with diminished overall survival, poor clinical outcome, and resistance of cancer cells to radiotherapy and chemotherapy [26]–[28], while the down-expression of Prx-I by RNAi is associated with therapeutic challenges for liver cancer, esophageal cancer, and thyroid cancer [29]–[31]. Actually, based on the iTRAQ analysis (Table S1), Western blot (Fig. 3A) and IHC analysis (Fig. 3B) in the present study, Prx-I expression significantly decreased in bladder cancer tissues after treatment with BI-TK/GCV, indicating that Prx-I may contribute to the effect of BI-TK/GCV treatment on anti-growth and pro-apoptosis of bladder cancer. Knockdown of Prx-I gene by shRNA significantly suppressed growth, promoted apoptosis and regulated the cell cycle in bladder cancer cells (Figure 6, 7). These results demonstrate the positive role of Prx-I in the development of bladder cancer. We presume that BI-TK/GCV treatment system is able to prevent tumor growth and induce apoptosis in the rodent bladder cancer model by down-regulating Prx-I expression. But what signaling pathway it is through?

Fortunately, the links between Prk-I and the NF-κB complex signaling have been implied in the protein pathway of IPA (Figure 2C), which lead us to find the clue. Prxs have been implicated as key regulatory factors in redox signaling [32]–[34]. Prxs can quench the second messenger H_2_O_2_ and inhibit signal transduction by activating the metabolism of H_2_O_2_. Overoxidation of Prx from a cysteine-sulfenic acid (Cys-SOH) to a cysteinesulfinic acid (Cys-SO_2_H) can stop the metabolism of H_2_O_2_, allowing the accumulation of H_2_O_2_ concentration and the propagation of signal transduction. Reduction of Cys-SO2H to Cys-SOH is achieved through the actions of sulfiredoxin, which restores Prx-mediated H_2_O_2_ metabolism [35]–[37]. Cytoplasmic Prx1 regulates H_2_O_2_-dependent NF-κB activation and nuclear translocation, and nuclear Prx1 regulates NF-κB/DNA binding through elimination of H_2_O_2_ as a p50 subunit oxidant [38]. Prx1 enhances p65-mediated cyclooxygenase (COX)-2 gene expression in estrogen receptor (ER) deficient human breast cancer cells (MDA-MB-231), and knockdown of Prx-I can attenuate COX-2 expression by reducing the occupancy of NF-κB at its upstream promoter element, indicating that Prx-I acts as a chaperone to enhance the transactivation potential of NF-kappaB in ER-deficient-breast cancer cells [39]. Actually, in the present study, knockdown of Prx-I reduced protein expressions of phospho-NF-κB p50 and p65, and thus it suppressed growth, promoted apoptosis and regulated the cell cycle of bladder cancer cells by inhibiting the NF-κB pathway, which conformed to the IPA network (Figure 2C).

## Conclusions

Taken together, we identified that Prx-I along with the NF-κB pathway contributed to bladder cancer for the first time. The BI-TK/GCV treatment system exhibited a sustainable anti-tumor growth activity and induced apoptosis in bladder cancer tissues by inhibition of Prx-I through the NF-κB pathway. Our research provides a new insight into bladder cancer treatment and indicates that BI-TK/GCV treatment system by targeting at Prx-I can be a novel therapeutic strategy in the future.

## Supporting Information

Table S1 **iTRAQ Analysis of Differentially Expressed Proteins in normal saline group (iTRAQ 114), BI-TK group (iTRAQ 115), BI/PGEX-1 group (iTRAQ 116) and BI group (iTRAQ 117).**
(DOCX)
