# Supplementary material for: Proteomic Analysis of Bladder Cancer Indicates Prx-I as a Key Molecule in BI-TK/GCV Treatment System
Source: PLoS One. 2014 Jun 6;9(6):e98764. doi: 10.1371/journal.pone.0098764 (PMC4048271; doi:10.1371/journal.pone.0098764)
Supplement: Table S1 — iTRAQ Analysis of Differentially Expressed Proteins in normal saline group (iTRAQ 114), BI-TK group (iTRAQ 115), BI/PGEX-1 group (iTRAQ 116) and BI group (iTRAQ 117). (DOCX) [file pone.0098764.s001.docx]

**Table S1. iTRAQ Analysis of Differentially Expressed Proteins in normal saline group(iTRAQ 114),BI-TK group(iTRAQ 115), BI/PGEX-1 group(iTRAQ 116 ) and BI group(iTRAQ 117).**

| Number | Accession # | Name | 115:114 | 116:114 | 117:114 |
| --- | --- | --- | --- | --- | --- |
|  |  |  |  |  |  |
| 1 | sp\|P30427\|PLEC_RAT | Plectin OS=Rattus norvegicus GN=Plec PE=1 SV=2 | 2.0701 | 1.6144 | 1.3932 |
| 2 | sp\|Q62812\|MYH9_RAT | Myosin-9 OS=Rattus norvegicus GN=Myh9 PE=1 SV=3 | 1.4997 | 1.0864 | 0.955 |
| 3 | sp\|P38650\|DYHC1_RAT | Cytoplasmic dynein 1 heavy chain 1 OS=Rattus norvegicus GN=Dync1h1 PE=1 SV=1 | 1.3474 | 1.406 | 1.1695 |
| 4 | sp\|P16086\|SPTN1_RAT | Spectrin alpha chain, non-erythrocytic 1 OS=Rattus norvegicus GN=Sptan1 PE=1 SV=2 | 1.9231 | 1.3305 | 1.1272 |
| 5 | sp\|Q63862\|MYH11_RAT | Myosin-11 (Fragments) OS=Rattus norvegicus GN=Myh11 PE=2 SV=3 | 0.4831 | 0.52 | 0.5916 |
| 6 | sp\|Q6P6Q2\|K2C5_RAT | Keratin, type II cytoskeletal 5 OS=Rattus norvegicus GN=Krt5 PE=1 SV=1 | 1.9638 | 4.1305 | 2.9648 |
| 7 | sp\|Q9Z1P2\|ACTN1_RAT | Alpha-actinin-1 OS=Rattus norvegicus GN=Actn1 PE=1 SV=1 | 0.5546 | 0.6026 | 0.6855 |
| 8 | sp\|P04937\|FINC_RAT | Fibronectin OS=Rattus norvegicus GN=Fn1 PE=1 SV=2 | 1.40093 | 1 | 1 |
| 9 | sp\|P01026\|CO3_RAT | Complement C3 OS=Rattus norvegicus GN=C3 PE=1 SV=3 | 2.7542 | 4.9659 | 2.8314 |
| 10 | sp\|P11442\|CLH_RAT | Clathrin heavy chain 1 OS=Rattus norvegicus GN=Cltc PE=1 SV=3 | 1.6144 | 1.7061 | 1.0568 |
| 11 | sp\|P02454\|CO1A1_RAT | Collagen alpha-1(I) chain OS=Rattus norvegicus GN=Col1a1 PE=1 SV=5 | 2.5586 | 0.0982 | 2.7542 |
| 12 | sp\|Q6IMF3\|K2C1_RAT | Keratin, type II cytoskeletal 1 OS=Rattus norvegicus GN=Krt1 PE=2 SV=1 | 0.5598 | 2.6062 | 3.6644 |
| 13 | sp\|P85972\|VINC_RAT | Vinculin OS=Rattus norvegicus GN=Vcl PE=1 SV=1 | 0.2704 | 0.4246 | 0.6026 |
| 14 | sp\|P02770\|ALBU_RAT | Serum albumin OS=Rattus norvegicus GN=Alb PE=1 SV=2 | 3.6308 | 4.0179 | 2.3335 |
| 15 | sp\|P48675\|DESM_RAT | Desmin OS=Rattus norvegicus GN=Des PE=1 SV=2 | 0.1941 | 0.2421 | 0.4875 |
| 16 | sp\|Q63041\|A1M_RAT | Alpha-1-macroglobulin OS=Rattus norvegicus GN=A1m PE=1 SV=1 | 2.4434 | 4.4055 | 2.3988 |
| 17 | sp\|P11980\|KPYM_RAT | Pyruvate kinase isozymes M1/M2 OS=Rattus norvegicus GN=Pkm PE=1 SV=3 | 0.631 | 0.8954 | 0.8954 |
| 18 | sp\|P11598\|PDIA3_RAT | Protein disulfide-isomerase A3 OS=Rattus norvegicus GN=Pdia3 PE=1 SV=2 | 1.4588 | 1.4859 | 1.2359 |
| 19 | sp\|P05197\|EF2_RAT | Elongation factor 2 OS=Rattus norvegicus GN=Eef2 PE=1 SV=4 | 1.5908 | 1.0093 | 0.9908 |
| 20 | sp\|Q6IFW6\|K1C10_RAT | Keratin, type I cytoskeletal 10 OS=Rattus norvegicus GN=Krt10 PE=2 SV=1 | 0.1754 | 2.5351 | 3.2509 |
| 21 | sp\|P48037\|ANXA6_RAT | Annexin A6 OS=Rattus norvegicus GN=Anxa6 PE=1 SV=2 | 1.4134 | 0.5649 | 0.929 |
| 22 | sp\|P12346\|TRFE_RAT | Serotransferrin OS=Rattus norvegicus GN=Tf PE=1 SV=3 | 2.3988 | 4.4463 | 3.3729 |
| 23 | sp\|P63018\|HSP7C_RAT | Heat shock cognate 71 kDa protein OS=Rattus norvegicus GN=Hspa8 PE=1 SV=1 | 0.6798 | 0.7047 | 0.6918 |
| 24 | sp\|P48679\|LMNA_RAT | Prelamin-A/C OS=Rattus norvegicus GN=Lmna PE=1 SV=1 | 1.6904 | 1.0186 | 1.1376 |
| 25 | sp\|P15800\|LAMB2_RAT | Laminin subunit beta-2 OS=Rattus norvegicus GN=Lamb2 PE=2 SV=1 | 1.5375 | 0.8954 | 0.6982 |
| 26 | sp\|P02466\|CO1A2_RAT | Collagen alpha-2(I) chain OS=Rattus norvegicus GN=Col1a2 PE=1 SV=3 | 1.7061 | 0.0353 | 2.1086 |
| 27 | sp\|P12785\|FAS_RAT | Fatty acid synthase OS=Rattus norvegicus GN=Fasn PE=1 SV=3 | 0.5872 | 1.3183 | 0.7798 |
| 28 | sp\|Q03626\|MUG1_RAT | Murinoglobulin-1 OS=Rattus norvegicus GN=Mug1 PE=2 SV=1 | 1.9409 | 1.6904 | 1.0093 |
| 29 | sp\|P62630\|EF1A1_RAT | Elongation factor 1-alpha 1 OS=Rattus norvegicus GN=Eef1a1 PE=1 SV=1 | 0.6607 | 0.7798 | 1 |
| 30 | sp\|Q6IFV3\|K1C15_RAT | Keratin, type I cytoskeletal 15 OS=Rattus norvegicus GN=Krt15 PE=1 SV=1 | 1.7066 | 1.2942 | 0.9638 |
| 31 | sp\|P53534\|PYGB_RAT | Glycogen phosphorylase, brain form (Fragment) OS=Rattus norvegicus GN=Pygb PE=1 SV=3 | 0.5754 | 0.6607 | 0.6792 |
| 32 | sp\|Q62667\|MVP_RAT | Major vault protein OS=Rattus norvegicus GN=Mvp PE=1 SV=4 | 0.579 | 0.5445 | 0.6546 |
| 33 | sp\|Q9ER34\|ACON_RAT | Aconitate hydratase, mitochondrial OS=Rattus norvegicus GN=Aco2 PE=1 SV=2 | 0.6017 | 1.1695 | 0.9036 |
| 34 | sp\|P07150\|ANXA1_RAT | Annexin A1 OS=Rattus norvegicus GN=Anxa1 PE=1 SV=2 | 0.3342 | 1 | 0.9204 |
| 35 | sp\|P69897\|TBB5_RAT | Tubulin beta-5 chain OS=Rattus norvegicus GN=Tubb5 PE=1 SV=1 | 1.3965 | 0.879 | 0.8954 |
| 36 | sp\|Q07936\|ANXA2_RAT | Annexin A2 OS=Rattus norvegicus GN=Anxa2 PE=1 SV=2 | 1.5849 | 1.8535 | 1.6596 |
| 37 | sp\|P63039\|CH60_RAT | 60 kDa heat shock protein, mitochondrial OS=Rattus norvegicus GN=Hspd1 PE=1 SV=1 | 1.529 | 1.4454 | 0.9462 |
| 38 | sp\|P06761\|GRP78_RAT | 78 kDa glucose-regulated protein OS=Rattus norvegicus GN=Hspa5 PE=1 SV=1 | 1.406 | 1.3677 | 1.3305 |
| 39 | sp\|Q4FZU2\|K2C6A_RAT | Keratin, type II cytoskeletal 6A OS=Rattus norvegicus GN=Krt6a PE=1 SV=1 | 1.3804 | 1.3428 | 3.3113 |
| 40 | sp\|P20059\|HEMO_RAT | Hemopexin OS=Rattus norvegicus GN=Hpx PE=1 SV=3 | 3.5975 | 6.0813 | 4.7424 |
| 41 | sp\|P14480\|FIBB_RAT | Fibrinogen beta chain OS=Rattus norvegicus GN=Fgb PE=1 SV=4 | 6.2517 | 8.091 | 5.1051 |
| 42 | sp\|P06685\|AT1A1_RAT | Sodium/potassium-transporting ATPase subunit alpha-1 OS=Rattus norvegicus GN=Atp1a1 PE=1 SV=1 | 1.879 | 1.2134 | 0.7379 |
| 43 | sp\|Q66HD0\|ENPL_RAT | Endoplasmin OS=Rattus norvegicus GN=Hsp90b1 PE=1 SV=2 | 1.6904 | 0.9727 | 1.0568 |
| 44 | sp\|P31000\|VIME_RAT | Vimentin OS=Rattus norvegicus GN=Vim PE=1 SV=2 | 2.4434 | 1.4454 | 1.5704 |
| 45 | sp\|P50399\|GDIB_RAT | Rab GDP dissociation inhibitor beta OS=Rattus norvegicus GN=Gdi2 PE=1 SV=2 | 0.629 | 0.9727 | 0.955 |
| 46 | sp\|Q9JLA3\|UGGG1_RAT | UDP-glucose:glycoprotein glucosyltransferase 1 OS=Rattus norvegicus GN=Uggt1 PE=1 SV=2 | 1.8197 | 1.1803 | 0.8318 |
| 47 | sp\|Q63279\|K1C19_RAT | Keratin, type I cytoskeletal 19 OS=Rattus norvegicus GN=Krt19 PE=1 SV=2 | 1.8707 | 2.1478 | 1.6749 |
| 48 | sp\|P10719\|ATPB_RAT | ATP synthase subunit beta, mitochondrial OS=Rattus norvegicus GN=Atp5b PE=1 SV=2 | 0.5765 | 0.879 | 0.6855 |
| 49 | sp\|Q6P9V9\|TBA1B_RAT | Tubulin alpha-1B chain OS=Rattus norvegicus GN=Tuba1b PE=1 SV=1 | 0.629 | 0.9817 | 0.9727 |
| 50 | sp\|Q4V8H8\|EHD2_RAT | EH domain-containing protein 2 OS=Rattus norvegicus GN=Ehd2 PE=1 SV=1 | 1.3912 | 0.8551 | 1.0666 |
| 51 | sp\|O88600\|HSP74_RAT | Heat shock 70 kDa protein 4 OS=Rattus norvegicus GN=Hspa4 PE=1 SV=1 | 0.6668 | 0.787 | 0.6792 |
| 52 | sp\|P04785\|PDIA1_RAT | Protein disulfide-isomerase OS=Rattus norvegicus GN=P4hb PE=1 SV=2 | 1.5849 | 1.4588 | 1.406 |
| 53 | sp\|P04797\|G3P_RAT | Glyceraldehyde-3-phosphate dehydrogenase OS=Rattus norvegicus GN=Gapdh PE=1 SV=3 | 0.2911 | 0.8551 | 0.6792 |
| 54 | sp\|Q66X93\|SND1_RAT | Staphylococcal nuclease domain-containing protein 1 OS=Rattus norvegicus GN=Snd1 PE=2 SV=1 | 1.4568 | 0.7798 | 0.8395 |
| 55 | sp\|P10860\|DHE3_RAT | Glutamate dehydrogenase 1, mitochondrial OS=Rattus norvegicus GN=Glud1 PE=1 SV=2 | 1.3305 | 1.1272 | 0.8166 |
| 56 | sp\|P04764\|ENOA_RAT | Alpha-enolase OS=Rattus norvegicus GN=Eno1 PE=1 SV=4 | 0.2858 | 1.028 | 0.7447 |
| 57 | sp\|Q68FQ0\|TCPE_RAT | T-complex protein 1 subunit epsilon OS=Rattus norvegicus GN=Cct5 PE=1 SV=1 | 0.5472 | 1.0471 | 0.5702 |
| 58 | sp\|P08649\|CO4_RAT | Complement C4 OS=Rattus norvegicus GN=C4 PE=1 SV=3 | 2.1281 | 1.7539 | 1.4191 |
| 59 | sp\|D3ZHV2\|MACF1_RAT | Microtubule-actin cross-linking factor 1 OS=Rattus norvegicus GN=Macf1 PE=1 SV=1 | 1.5643 | 0.9376 | 0.9204 |
| 60 | sp\|P00507\|AATM_RAT | Aspartate aminotransferase, mitochondrial OS=Rattus norvegicus GN=Got2 PE=1 SV=2 | 1.4638 | 0.9908 | 0.9462 |
| 61 | sp\|Q9JLT0\|MYH10_RAT | Myosin-10 OS=Rattus norvegicus GN=Myh10 PE=1 SV=1 | 0.679 | 0.912 | 1.028 |
| 62 | sp\|Q5SGE0\|LPPRC_RAT | Leucine-rich PPR motif-containing protein, mitochondrial OS=Rattus norvegicus GN=Lrpprc PE=1 SV=1 | 1.6376 | 1.0568 | 1.0666 |
| 63 | sp\|Q9Z2Q1\|SC31A_RAT | Protein transport protein Sec31A OS=Rattus norvegicus GN=Sec31a PE=1 SV=2 | 1.5028 | 0.9908 | 0.9908 |
| 64 | sp\|Q10758\|K2C8_RAT | Keratin, type II cytoskeletal 8 OS=Rattus norvegicus GN=Krt8 PE=1 SV=3 | 1.8535 | 1.6444 | 1.6749 |
| 65 | sp\|P05708\|HXK1_RAT | Hexokinase-1 OS=Rattus norvegicus GN=Hk1 PE=1 SV=4 | 0.6368 | 0.9376 | 0.8472 |
| 66 | sp\|P01946\|HBA_RAT | Hemoglobin subunit alpha-1/2 OS=Rattus norvegicus GN=Hba1 PE=1 SV=3 | 2.1588 | 4.9659 | 1.803 |
| 67 | sp\|P08932\|KNT2_RAT | T-kininogen 2 OS=Rattus norvegicus PE=1 SV=2 | 2.2284 | 4.1687 | 3.1623 |
| 68 | sp\|Q9QXQ0\|ACTN4_RAT | Alpha-actinin-4 OS=Rattus norvegicus GN=Actn4 PE=1 SV=2 | 0.6668 | 0.863 | 0.7178 |
| 69 | sp\|P50137\|TKT_RAT | Transketolase OS=Rattus norvegicus GN=Tkt PE=1 SV=1 | 1.9375 | 1.5704 | 1.3677 |
| 70 | sp\|Q5RKI0\|WDR1_RAT | WD repeat-containing protein 1 OS=Rattus norvegicus GN=Wdr1 PE=1 SV=3 | 0.5861 | 0.7943 | 0.6982 |
| 71 | sp\|Q63598\|PLST_RAT | Plastin-3 OS=Rattus norvegicus GN=Pls3 PE=2 SV=2 | 1.7965 | 1.3305 | 1.803 |
| 72 | sp\|P35559\|IDE_RAT | Insulin-degrading enzyme OS=Rattus norvegicus GN=Ide PE=1 SV=1 | 0.4365 | 1.4191 | 1.6293 |
| 73 | sp\|P05370\|G6PD_RAT | Glucose-6-phosphate 1-dehydrogenase OS=Rattus norvegicus GN=G6pdx PE=1 SV=3 | 1.8241 | 1.3183 | 1.1376 |
| 74 | sp\|Q63355\|MYO1C_RAT | Unconventional myosin-Ic OS=Rattus norvegicus GN=Myo1c PE=2 SV=2 | 1.4375 | 0.871 | 0.9462 |
| 75 | sp\|Q64428\|ECHA_RAT | Trifunctional enzyme subunit alpha, mitochondrial OS=Rattus norvegicus GN=Hadha PE=1 SV=2 | 1.4246 | 1.1482 | 1.1588 |
| 76 | sp\|P02680\|FIBG_RAT | Fibrinogen gamma chain OS=Rattus norvegicus GN=Fgg PE=1 SV=3 | 16.4437 | 15.7036 | 10.5682 |
| 77 | sp\|P52873\|PYC_RAT | Pyruvate carboxylase, mitochondrial OS=Rattus norvegicus GN=Pc PE=1 SV=2 | 1.7061 | 2.3988 | 1.5704 |
| 78 | sp\|Q63617\|HYOU1_RAT | Hypoxia up-regulated protein 1 OS=Rattus norvegicus GN=Hyou1 PE=1 SV=1 | 1.5645 | 1.1912 | 1.2134 |
| 79 | sp\|P16617\|PGK1_RAT | Phosphoglycerate kinase 1 OS=Rattus norvegicus GN=Pgk1 PE=1 SV=2 | 0.5058 | 1.2589 | 1.1169 |
| 80 | sp\|Q6P0K8\|PLAK_RAT | Junction plakoglobin OS=Rattus norvegicus GN=Jup PE=1 SV=1 | 1.4765 | 1.2706 | 2.0512 |
| 81 | sp\|P06399\|FIBA_RAT | Fibrinogen alpha chain OS=Rattus norvegicus GN=Fga PE=1 SV=3 | 7.9433 | 7.8705 | 5.5976 |
| 82 | sp\|Q01177\|PLMN_RAT | Plasminogen OS=Rattus norvegicus GN=Plg PE=2 SV=2 | 3.4674 | 4.0179 | 3.0761 |
| 83 | sp\|P97571\|CAN1_RAT | Calpain-1 catalytic subunit OS=Rattus norvegicus GN=Capn1 PE=1 SV=1 | 1.5359 | 0.9204 | 0.9462 |
| 84 | sp\|P47860\|K6PP_RAT | 6-phosphofructokinase type C OS=Rattus norvegicus GN=Pfkp PE=1 SV=2 | 0.6918 | 1.2134 | 1.4588 |
| 85 | sp\|Q641Y8\|DDX1_RAT | ATP-dependent RNA helicase DDX1 OS=Rattus norvegicus GN=Ddx1 PE=2 SV=1 | 1.3589 | 1.3062 | 1.3183 |
| 86 | sp\|P07335\|KCRB_RAT | Creatine kinase B-type OS=Rattus norvegicus GN=Ckb PE=1 SV=2 | 0.5546 | 0.7586 | 0.8318 |
| 87 | sp\|Q02874\|H2AY_RAT | Core histone macro-H2A.1 OS=Rattus norvegicus GN=H2afy PE=1 SV=4 | 1.6023 | 0.6081 | 0.871 |
| 88 | sp\|Q5XI78\|ODO1_RAT | 2-oxoglutarate dehydrogenase, mitochondrial OS=Rattus norvegicus GN=Ogdh PE=1 SV=1 | 1.7539 | 1.1272 | 0.7311 |
| 89 | sp\|Q6IG00\|K2C4_RAT | Keratin, type II cytoskeletal 4 OS=Rattus norvegicus GN=Krt4 PE=2 SV=1 | 0.3017 | 0.2965 | 0.7047 |
| 90 | sp\|P18484\|AP2A2_RAT | AP-2 complex subunit alpha-2 OS=Rattus norvegicus GN=Ap2a2 PE=1 SV=3 | 1.4588 | 1.1695 | 1.0666 |
| 91 | sp\|Q62826\|HNRPM_RAT | Heterogeneous nuclear ribonucleoprotein M OS=Rattus norvegicus GN=Hnrnpm PE=1 SV=4 | 1.4093 | 0.9817 | 0.9727 |
| 92 | sp\|Q10728\|MYPT1_RAT | Protein phosphatase 1 regulatory subunit 12A OS=Rattus norvegicus GN=Ppp1r12a PE=1 SV=2 | 0.6379 | 0.673 | 0.7943 |
| 93 | sp\|P48721\|GRP75_RAT | Stress-70 protein, mitochondrial OS=Rattus norvegicus GN=Hspa9 PE=1 SV=3 | 0.5318 | 1.1376 | 0.7244 |
| 94 | sp\|Q9WTT6\|GUAD_RAT | Guanine deaminase OS=Rattus norvegicus GN=Gda PE=1 SV=1 | 1.5996 | 2.5351 | 2.704 |
| 95 | sp\|P11884\|ALDH2_RAT | Aldehyde dehydrogenase, mitochondrial OS=Rattus norvegicus GN=Aldh2 PE=1 SV=1 | 0.492 | 0.6982 | 0.4699 |
| 96 | sp\|Q6P502\|TCPG_RAT | T-complex protein 1 subunit gamma OS=Rattus norvegicus GN=Cct3 PE=1 SV=1 | 0.6447 | 0.7798 | 0.7727 |
| 97 | sp\|P07153\|RPN1_RAT | Dolichyl-diphosphooligosaccharide--protein glycosyltransferase subunit 1 OS=Rattus norvegicus GN=Rpn1 PE=2 SV=1 | 1.6749 | 1.1803 | 1.1588 |
| 98 | sp\|P56574\|IDHP_RAT | Isocitrate dehydrogenase [NADP], mitochondrial OS=Rattus norvegicus GN=Idh2 PE=1 SV=2 | 0.6855 | 1.0375 | 0.9462 |
| 99 | sp\|Q75WE7\|VWA5A_RAT | von Willebrand factor A domain-containing protein 5A OS=Rattus norvegicus GN=Vwa5a PE=2 SV=1 | 0.5586 | 0.6918 | 0.5297 |
| 100 | sp\|P16638\|ACLY_RAT | ATP-citrate synthase OS=Rattus norvegicus GN=Acly PE=1 SV=1 | 0.6727 | 1 | 0.9817 |
| 101 | sp\|P70615\|LMNB1_RAT | Lamin-B1 OS=Rattus norvegicus GN=Lmnb1 PE=1 SV=3 | 1.3305 | 0.8395 | 0.7727 |
| 102 | sp\|P13635\|CERU_RAT | Ceruloplasmin OS=Rattus norvegicus GN=Cp PE=1 SV=3 | 2.6792 | 3.8726 | 2.8576 |
| 103 | sp\|P20760\|IGG2A_RAT | Ig gamma-2A chain C region OS=Rattus norvegicus GN=Igg-2a PE=1 SV=1 | 4.6989 | 6.3096 | 3.8726 |
| 104 | sp\|P62909\|RS3_RAT | 40S ribosomal protein S3 OS=Rattus norvegicus GN=Rps3 PE=2 SV=1 | 1.6093 | 0.9908 | 1.0471 |
| 105 | sp\|P63245\|GBLP_RAT | Guanine nucleotide-binding protein subunit beta-2-like 1 OS=Rattus norvegicus GN=Gnb2l1 PE=1 SV=3 | 1.7093 | 1.0093 | 1.0093 |
| 106 | sp\|P14669\|ANXA3_RAT | Annexin A3 OS=Rattus norvegicus GN=Anxa3 PE=1 SV=4 | 0.5861 | 0.9376 | 0.8091 |
| 107 | sp\|P29457\|SERPH_RAT | Serpin H1 OS=Rattus norvegicus GN=Serpinh1 PE=1 SV=1 | 1.3638 | 0.9817 | 0.9908 |
| 108 | sp\|P04692\|TPM1_RAT | Tropomyosin alpha-1 chain OS=Rattus norvegicus GN=Tpm1 PE=1 SV=3 | 0.6026 | 0.5445 | 0.9727 |
| 109 | sp\|Q9EPH8\|PABP1_RAT | Polyadenylate-binding protein 1 OS=Rattus norvegicus GN=Pabpc1 PE=2 SV=1 | 0.5395 | 0.7586 | 0.9204 |
| 110 | sp\|Q5XI07\|LPP_RAT | Lipoma-preferred partner homolog OS=Rattus norvegicus GN=Lpp PE=1 SV=1 | 0.4699 | 0.4325 | 0.5598 |
| 111 | sp\|P47942\|DPYL2_RAT | Dihydropyrimidinase-related protein 2 OS=Rattus norvegicus GN=Dpysl2 PE=1 SV=1 | 1.5471 | 1.1588 | 1.2589 |
| 112 | sp\|Q68FP1\|GELS_RAT | Gelsolin OS=Rattus norvegicus GN=Gsn PE=1 SV=1 | 1.3942 | 1.0864 | 1.0765 |
| 113 | sp\|P62944\|AP2B1_RAT | AP-2 complex subunit beta OS=Rattus norvegicus GN=Ap2b1 PE=1 SV=1 | 1.6596 | 1.3804 | 1.0765 |
| 114 | sp\|P32198\|CPT1A_RAT | Carnitine O-palmitoyltransferase 1, liver isoform OS=Rattus norvegicus GN=Cpt1a PE=1 SV=2 | 1.6965 | 1.6293 | 1.5136 |
| 115 | sp\|P10960\|SAP_RAT | Sulfated glycoprotein 1 OS=Rattus norvegicus GN=Psap PE=1 SV=1 | 0.6607 | 1.6293 | 1.3305 |
| 116 | sp\|P31232\|TAGL_RAT | Transgelin OS=Rattus norvegicus GN=Tagln PE=1 SV=2 | 0.169 | 0.5495 | 0.7047 |
| 117 | sp\|Q7TPB1\|TCPD_RAT | T-complex protein 1 subunit delta OS=Rattus norvegicus GN=Cct4 PE=1 SV=3 | 0.5472 | 1.1695 | 0.7656 |
| 118 | sp\|O35567\|PUR9_RAT | Bifunctional purine biosynthesis protein PURH OS=Rattus norvegicus GN=Atic PE=1 SV=2 | 0.5808 | 1.0864 | 0.9817 |
| 119 | sp\|Q1JU68\|EIF3A_RAT | Eukaryotic translation initiation factor 3 subunit A OS=Rattus norvegicus GN=Eif3a PE=2 SV=2 | 0.6836 | 0.871 | 0.6427 |
| 120 | sp\|Q4G075\|ILEUA_RAT | Leukocyte elastase inhibitor A OS=Rattus norvegicus GN=Serpinb1a PE=2 SV=1 | 0.6747 | 0.787 | 0.8395 |
| 121 | sp\|P04905\|GSTM1_RAT | Glutathione S-transferase Mu 1 OS=Rattus norvegicus GN=Gstm1 PE=1 SV=2 | 0.4285 | 0.6982 | 1.0864 |
| 122 | sp\|P13832\|MRLCA_RAT | Myosin regulatory light chain RLC-A OS=Rattus norvegicus GN=Rlc-a PE=2 SV=2 | 1.3183 | 1.1482 | 1.2359 |
| 123 | sp\|B5DFC9\|NID2_RAT | Nidogen-2 OS=Rattus norvegicus GN=Nid2 PE=2 SV=1 | 1.564 | 0.9462 | 0.9817 |
| 124 | sp\|P38659\|PDIA4_RAT | Protein disulfide-isomerase A4 OS=Rattus norvegicus GN=Pdia4 PE=1 SV=2 | 1.5471 | 0.9908 | 0.9817 |
| 125 | sp\|P85968\|6PGD_RAT | 6-phosphogluconate dehydrogenase, decarboxylating OS=Rattus norvegicus GN=Pgd PE=1 SV=1 | 0.3945 | 0.8551 | 0.955 |
| 126 | sp\|Q9EQP5\|PRELP_RAT | Prolargin OS=Rattus norvegicus GN=Prelp PE=2 SV=1 | 1.6904 | 0.7586 | 1.406 |
| 127 | sp\|Q9QWN8\|SPTN2_RAT | Spectrin beta chain, non-erythrocytic 2 OS=Rattus norvegicus GN=Sptbn2 PE=1 SV=2 | 1.4375 | 0.9462 | 0.9908 |
| 128 | sp\|P22985\|XDH_RAT | Xanthine dehydrogenase/oxidase OS=Rattus norvegicus GN=Xdh PE=1 SV=3 | 0.6727 | 0.955 | 0.9817 |
| 129 | sp\|P05065\|ALDOA_RAT | Fructose-bisphosphate aldolase A OS=Rattus norvegicus GN=Aldoa PE=1 SV=2 | 0.6138 | 1.0965 | 0.863 |
| 130 | sp\|Q4FZT9\|PSMD2_RAT | 26S proteasome non-ATPase regulatory subunit 2 OS=Rattus norvegicus GN=Psmd2 PE=2 SV=1 | 0.6727 | 0.879 | 0.8395 |
| 131 | sp\|Q6URK4\|ROA3_RAT | Heterogeneous nuclear ribonucleoprotein A3 OS=Rattus norvegicus GN=Hnrnpa3 PE=1 SV=1 | 0.6516 | 1.1588 | 1 |
| 132 | sp\|Q5BJY9\|K1C18_RAT | Keratin, type I cytoskeletal 18 OS=Rattus norvegicus GN=Krt18 PE=1 SV=3 | 2.1086 | 1.8365 | 1.7378 |
| 133 | sp\|P35565\|CALX_RAT | Calnexin OS=Rattus norvegicus GN=Canx PE=1 SV=1 | 1.5568 | 0.9908 | 0.9817 |
| 134 | sp\|P23514\|COPB_RAT | Coatomer subunit beta OS=Rattus norvegicus GN=Copb1 PE=1 SV=1 | 1.3305 | 1.1588 | 0.8954 |
| 135 | sp\|Q07439\|HSP71_RAT | Heat shock 70 kDa protein 1A/1B OS=Rattus norvegicus GN=Hspa1a PE=2 SV=2 | 0.5598 | 0.5445 | 0.6792 |
| 136 | sp\|P04636\|MDHM_RAT | Malate dehydrogenase, mitochondrial OS=Rattus norvegicus GN=Mdh2 PE=1 SV=2 | 0.6241 | 1.1803 | 0.787 |
| 137 | sp\|P49432\|ODPB_RAT | Pyruvate dehydrogenase E1 component subunit beta, mitochondrial OS=Rattus norvegicus GN=Pdhb PE=1 SV=2 | 0.671 | 1.6293 | 1.0186 |
| 138 | sp\|P51647\|AL1A1_RAT | Retinal dehydrogenase 1 OS=Rattus norvegicus GN=Aldh1a1 PE=1 SV=3 | 2.355 | 2.729 | 1.6444 |
| 139 | sp\|Q2PQA9\|KINH_RAT | Kinesin-1 heavy chain OS=Rattus norvegicus GN=Kif5b PE=2 SV=1 | 1.3638 | 0.9204 | 0.955 |
| 140 | sp\|P17475\|A1AT_RAT | Alpha-1-antiproteinase OS=Rattus norvegicus GN=Serpina1 PE=1 SV=2 | 1.4322 | 1.7701 | 1.3932 |
| 141 | sp\|P52296\|IMB1_RAT | Importin subunit beta-1 OS=Rattus norvegicus GN=Kpnb1 PE=1 SV=1 | 0.6241 | 1.0375 | 0.6918 |
| 142 | sp\|P62260\|1433E_RAT | 14-3-3 protein epsilon OS=Rattus norvegicus GN=Ywhae PE=1 SV=1 | 1.4727 | 0.9817 | 0.9908 |
| 143 | sp\|Q68FS4\|AMPL_RAT | Cytosol aminopeptidase OS=Rattus norvegicus GN=Lap3 PE=1 SV=1 | 1.479 | 1.0186 | 0.9727 |
| 144 | sp\|Q64632\|ITB4_RAT | Integrin beta-4 OS=Rattus norvegicus GN=Itgb4 PE=2 SV=1 | 1.879 | 1.3305 | 1.1066 |
| 145 | sp\|P09006\|SPA3N_RAT | Serine protease inhibitor A3N OS=Rattus norvegicus GN=Serpina3n PE=1 SV=3 | 2.9107 | 7.379 | 4.2855 |
| 146 | sp\|Q08163\|CAP1_RAT | Adenylyl cyclase-associated protein 1 OS=Rattus norvegicus GN=Cap1 PE=1 SV=3 | 0.6855 | 1.0765 | 0.8954 |
| 147 | sp\|Q63270\|ACOC_RAT | Cytoplasmic aconitate hydratase OS=Rattus norvegicus GN=Aco1 PE=1 SV=1 | 1.5093 | 0.9817 | 0.9727 |
| 148 | sp\|P04642\|LDHA_RAT | L-lactate dehydrogenase A chain OS=Rattus norvegicus GN=Ldha PE=1 SV=1 | 0.3467 | 0.9638 | 0.7178 |
| 149 | sp\|P10111\|PPIA_RAT | Peptidyl-prolyl cis-trans isomerase A OS=Rattus norvegicus GN=Ppia PE=1 SV=2 | 0.3945 | 1.0965 | 1.0965 |
| 150 | sp\|Q9QZA2\|PDC6I_RAT | Programmed cell death 6-interacting protein OS=Rattus norvegicus GN=Pdcd6ip PE=1 SV=2 | 1.528 | 1.0375 | 1.0093 |
| 151 | sp\|Q05962\|ADT1_RAT | ADP/ATP translocase 1 OS=Rattus norvegicus GN=Slc25a4 PE=1 SV=3 | 0.6727 | 0.8241 | 1.0765 |
| 152 | sp\|P97852\|DHB4_RAT | Peroxisomal multifunctional enzyme type 2 OS=Rattus norvegicus GN=Hsd17b4 PE=1 SV=3 | 1.4482 | 0.9727 | 0.7447 |
| 153 | sp\|Q6IG12\|K2C7_RAT | Keratin, type II cytoskeletal 7 OS=Rattus norvegicus GN=Krt7 PE=2 SV=1 | 2.208 | 1.7378 | 1.6749 |
| 154 | sp\|Q5XHZ0\|TRAP1_RAT | Heat shock protein 75 kDa, mitochondrial OS=Rattus norvegicus GN=Trap1 PE=1 SV=1 | 0.638 | 1.028 | 1 |
| 155 | sp\|Q66HF1\|NDUS1_RAT | NADH-ubiquinone oxidoreductase 75 kDa subunit, mitochondrial OS=Rattus norvegicus GN=Ndufs1 PE=1 SV=1 | 1.7093 | 0.9727 | 0.9817 |
| 156 | sp\|P13383\|NUCL_RAT | Nucleolin OS=Rattus norvegicus GN=Ncl PE=1 SV=3 | 0.479 | 1.1272 | 1.3062 |
| 157 | sp\|Q9QZR6\|SEPT9_RAT | Septin-9 OS=Rattus norvegicus GN=Sept9 PE=1 SV=1 | 0.6551 | 1.0186 | 1.2474 |
| 158 | sp\|Q6P6V0\|G6PI_RAT | Glucose-6-phosphate isomerase OS=Rattus norvegicus GN=Gpi PE=1 SV=1 | 0.5808 | 1.0666 | 0.8954 |
| 159 | sp\|P04762\|CATA_RAT | Catalase OS=Rattus norvegicus GN=Cat PE=1 SV=3 | 1.3817 | 0.9908 | 1.0375 |
| 160 | sp\|P0C219\|SLMAP_RAT | Sarcolemmal membrane-associated protein OS=Rattus norvegicus GN=Slmap PE=3 SV=1 | 0.3373 | 0.5808 | 0.6138 |
| 161 | sp\|P02091\|HBB1_RAT | Hemoglobin subunit beta-1 OS=Rattus norvegicus GN=Hbb PE=1 SV=3 | 1.9055 | 6.0813 | 2.7797 |
| 162 | sp\|P10760\|SAHH_RAT | Adenosylhomocysteinase OS=Rattus norvegicus GN=Ahcy PE=1 SV=3 | 0.5551 | 0.5861 | 0.6138 |
| 163 | sp\|P24268\|CATD_RAT | Cathepsin D OS=Rattus norvegicus GN=Ctsd PE=1 SV=1 | 0.3565 | 0.9727 | 0.5248 |
| 164 | sp\|Q64633\|UD17_RAT | UDP-glucuronosyltransferase 1-7 OS=Rattus norvegicus GN=Ugt1a7c PE=2 SV=1 | 2.6546 | 2.2491 | 2.0701 |
| 165 | sp\|Q4V7C7\|ARP3_RAT | Actin-related protein 3 OS=Rattus norvegicus GN=Actr3 PE=1 SV=1 | 0.6368 | 0.8395 | 0.8017 |
| 166 | sp\|P09650\|MCPT1_RAT | Mast cell protease 1 OS=Rattus norvegicus GN=Mcpt1 PE=1 SV=3 | 1.4359 | 0.2421 | 0.9204 |
| 167 | sp\|P04906\|GSTP1_RAT | Glutathione S-transferase P OS=Rattus norvegicus GN=Gstp1 PE=1 SV=2 | 0.5598 | 0.5248 | 0.5916 |
| 168 | sp\|Q08290\|CNN1_RAT | Calponin-1 OS=Rattus norvegicus GN=Cnn1 PE=2 SV=1 | 0.2089 | 0.2466 | 0.5598 |
| 169 | sp\|P29315\|RINI_RAT | Ribonuclease inhibitor OS=Rattus norvegicus GN=Rnh1 PE=1 SV=2 | 0.673 | 1.0471 | 0.9376 |
| 170 | sp\|P17764\|THIL_RAT | Acetyl-CoA acetyltransferase, mitochondrial OS=Rattus norvegicus GN=Acat1 PE=1 SV=1 | 1.6186 | 1 | 0.955 |
| 171 | sp\|O35142\|COPB2_RAT | Coatomer subunit beta' OS=Rattus norvegicus GN=Copb2 PE=1 SV=3 | 1.3471 | 1.0375 | 0.9727 |
| 172 | sp\|Q9QUL6\|NSF_RAT | Vesicle-fusing ATPase OS=Rattus norvegicus GN=Nsf PE=1 SV=1 | 0.66638 | 0.9908 | 0.9462 |
| 173 | sp\|P51635\|AK1A1_RAT | Alcohol dehydrogenase [NADP(+)] OS=Rattus norvegicus GN=Akr1a1 PE=1 SV=2 | 0.6792 | 0.6918 | 0.9204 |
| 174 | sp\|Q9ESN0\|NIBAN_RAT | Protein Niban OS=Rattus norvegicus GN=Fam129a PE=2 SV=2 | 0.6368 | 0.7447 | 0.912 |
| 175 | sp\|O35814\|STIP1_RAT | Stress-induced-phosphoprotein 1 OS=Rattus norvegicus GN=Stip1 PE=1 SV=1 | 0.5105 | 0.7244 | 0.5445 |
| 176 | sp\|P85973\|PNPH_RAT | Purine nucleoside phosphorylase OS=Rattus norvegicus GN=Pnp PE=1 SV=1 | 0.6166 | 0.6855 | 0.6918 |
| 177 | sp\|Q63797\|PSME1_RAT | Proteasome activator complex subunit 1 OS=Rattus norvegicus GN=Psme1 PE=2 SV=1 | 1.3817 | 1.0186 | 0.9204 |
| 178 | sp\|P49134\|ITB1_RAT | Integrin beta-1 OS=Rattus norvegicus GN=Itgb1 PE=2 SV=1 | 0.655 | 0.9638 | 0.929 |
| 179 | sp\|P50475\|SYAC_RAT | Alanine--tRNA ligase, cytoplasmic OS=Rattus norvegicus GN=Aars PE=1 SV=3 | 0.4446 | 0.6668 | 0.7244 |
| 180 | sp\|P32551\|QCR2_RAT | Cytochrome b-c1 complex subunit 2, mitochondrial OS=Rattus norvegicus GN=Uqcrc2 PE=1 SV=2 | 1.3066 | 0.955 | 0.8472 |
| 181 | sp\|Q62736\|CALD1_RAT | Non-muscle caldesmon OS=Rattus norvegicus GN=Cald1 PE=1 SV=1 | 0.6138 | 0.597 | 0.8241 |
| 182 | sp\|P14141\|CAH3_RAT | Carbonic anhydrase 3 OS=Rattus norvegicus GN=Ca3 PE=1 SV=3 | 10.8643 | 5.1523 | 2.355 |
| 183 | sp\|P50878\|RL4_RAT | 60S ribosomal protein L4 OS=Rattus norvegicus GN=Rpl4 PE=1 SV=3 | 1.6293 | 0.5495 | 1.7865 |
| 184 | sp\|Q63716\|PRDX1_RAT | Peroxiredoxin-1 OS=Rattus norvegicus GN=Prdx1 PE=1 SV=1 | 0.52 | 0.879 | 0.9036 |
| 185 | sp\|Q07009\|CAN2_RAT | Calpain-2 catalytic subunit OS=Rattus norvegicus GN=Capn2 PE=1 SV=3 | 0.631 | 0.912 | 0.6982 |
| 186 | sp\|P06866\|HPT_RAT | Haptoglobin OS=Rattus norvegicus GN=Hp PE=1 SV=3 | 7.1779 | 17.7011 | 11.9124 |
| 187 | sp\|Q01129\|PGS2_RAT | Decorin OS=Rattus norvegicus GN=Dcn PE=1 SV=1 | 1.3942 | 0.7447 | 1.6904 |
| 188 | sp\|Q7M0E3\|DEST_RAT | Destrin OS=Rattus norvegicus GN=Dstn PE=1 SV=3 | 0.2911 | 0.6546 | 0.9908 |
| 189 | sp\|P51886\|LUM_RAT | Lumican OS=Rattus norvegicus GN=Lum PE=2 SV=1 | 2.729 | 1.0093 | 1.5704 |
| 190 | sp\|P47853\|PGS1_RAT | Biglycan OS=Rattus norvegicus GN=Bgn PE=2 SV=1 | 0.3221 | 0.3373 | 1.3677 |
| 191 | sp\|Q64119\|MYL6_RAT | Myosin light polypeptide 6 OS=Rattus norvegicus GN=Myl6 PE=1 SV=3 | 0.6081 | 0.4786 | 0.6607 |
| 192 | sp\|Q3KR86\|IMMT_RAT | Mitochondrial inner membrane protein (Fragment) OS=Rattus norvegicus GN=Immt PE=1 SV=1 | 1.5849 | 1.9231 | 1.4454 |
| 193 | sp\|P41682\|ADH7_RAT | Alcohol dehydrogenase class 4 mu/sigma chain OS=Rattus norvegicus GN=Adh7 PE=1 SV=2 | 1.4588 | 3.2211 | 2.9107 |
| 194 | sp\|A7VJC2\|ROA2_RAT | Heterogeneous nuclear ribonucleoproteins A2/B1 OS=Rattus norvegicus GN=Hnrnpa2b1 PE=1 SV=1 | 0.5861 | 0.7798 | 0.8241 |
| 195 | sp\|P15650\|ACADL_RAT | Long-chain specific acyl-CoA dehydrogenase, mitochondrial OS=Rattus norvegicus GN=Acadl PE=1 SV=1 | 1.4588 | 0.8472 | 0.9462 |
| 196 | sp\|Q7TP47\|HNRPQ_RAT | Heterogeneous nuclear ribonucleoprotein Q OS=Rattus norvegicus GN=Syncrip PE=2 SV=1 | 0.6546 | 1.0471 | 1.2706 |
| 197 | sp\|Q6IFV4\|K1C13_RAT | Keratin, type I cytoskeletal 13 OS=Rattus norvegicus GN=Krt13 PE=2 SV=1 | 7.8705 | 13.1826 | 21.2814 |
| 198 | sp\|Q64057\|AL7A1_RAT | Alpha-aminoadipic semialdehyde dehydrogenase OS=Rattus norvegicus GN=Aldh7a1 PE=1 SV=2 | 1.5849 | 1.4997 | 1.3804 |
| 199 | sp\|P48500\|TPIS_RAT | Triosephosphate isomerase OS=Rattus norvegicus GN=Tpi1 PE=1 SV=2 | 0.5346 | 0.929 | 0.8872 |
| 200 | sp\|Q64542\|AT2B4_RAT | Plasma membrane calcium-transporting ATPase 4 OS=Rattus norvegicus GN=Atp2b4 PE=2 SV=1 | 0.6081 | 0.5861 | 0.7112 |
| 201 | sp\|O55096\|DPP3_RAT | Dipeptidyl peptidase 3 OS=Rattus norvegicus GN=Dpp3 PE=1 SV=2 | 0.6427 | 1.0765 | 1.2706 |
| 202 | sp\|Q9WUH4\|FHL1_RAT | Four and a half LIM domains protein 1 OS=Rattus norvegicus GN=Fhl1 PE=2 SV=1 | 0.2399 | 1.028 | 1.1376 |
| 203 | sp\|Q9EPF2\|MUC18_RAT | Cell surface glycoprotein MUC18 OS=Rattus norvegicus GN=Mcam PE=1 SV=2 | 0.6727 | 1.3062 | 1.028 |
| 204 | sp\|Q3MIE4\|VAT1_RAT | Synaptic vesicle membrane protein VAT-1 homolog OS=Rattus norvegicus GN=Vat1 PE=1 SV=1 | 1.3932 | 0.9462 | 1.2823 |
| 205 | sp\|P47875\|CSRP1_RAT | Cysteine and glycine-rich protein 1 OS=Rattus norvegicus GN=Csrp1 PE=2 SV=2 | 0.1282 | 0.8017 | 0.9462 |
| 206 | sp\|Q63416\|ITIH3_RAT | Inter-alpha-trypsin inhibitor heavy chain H3 OS=Rattus norvegicus GN=Itih3 PE=2 SV=1 | 1.5704 | 2.0701 | 4.8306 |
| 207 | sp\|P0C5E3\|PALLD_RAT | Palladin (Fragment) OS=Rattus norvegicus GN=Palld PE=2 SV=1 | 1.4997 | 0.9462 | 1.2134 |
| 208 | sp\|P45592\|COF1_RAT | Cofilin-1 OS=Rattus norvegicus GN=Cfl1 PE=1 SV=3 | 0.4875 | 0.9376 | 1.2359 |
| 209 | sp\|P20070\|NB5R3_RAT | NADH-cytochrome b5 reductase 3 OS=Rattus norvegicus GN=Cyb5r3 PE=1 SV=2 | 1.7865 | 1.4997 | 1.2706 |
| 210 | sp\|P82995\|HS90A_RAT | Heat shock protein HSP 90-alpha OS=Rattus norvegicus GN=Hsp90aa1 PE=1 SV=3 | 0.631 | 0.4875 | 0.5058 |
| 211 | sp\|Q63081\|PDIA6_RAT | Protein disulfide-isomerase A6 OS=Rattus norvegicus GN=Pdia6 PE=1 SV=2 | 1.5276 | 1.1912 | 1.1272 |
| 212 | sp\|O89049\|TRXR1_RAT | Thioredoxin reductase 1, cytoplasmic OS=Rattus norvegicus GN=Txnrd1 PE=1 SV=5 | 1.3804 | 4.5709 | 2.3335 |
| 213 | sp\|P04276\|VTDB_RAT | Vitamin D-binding protein OS=Rattus norvegicus GN=Gc PE=1 SV=3 | 1.4322 | 2.1878 | 1.7539 |
| 214 | sp\|P62963\|PROF1_RAT | Profilin-1 OS=Rattus norvegicus GN=Pfn1 PE=1 SV=2 | 0.5152 | 0.7586 | 0.7244 |
| 215 | sp\|P27952\|RS2_RAT | 40S ribosomal protein S2 OS=Rattus norvegicus GN=Rps2 PE=1 SV=1 | 1.3062 | 0.7516 | 1.1912 |
| 216 | sp\|P49242\|RS3A_RAT | 40S ribosomal protein S3a OS=Rattus norvegicus GN=Rps3a PE=1 SV=2 | 1.3428 | 0.7244 | 1.1588 |
| 217 | sp\|Q68FU3\|ETFB_RAT | Electron transfer flavoprotein subunit beta OS=Rattus norvegicus GN=Etfb PE=2 SV=3 | 0.6166 | 1.3552 | 1.1272 |
| 218 | sp\|O88989\|MDHC_RAT | Malate dehydrogenase, cytoplasmic OS=Rattus norvegicus GN=Mdh1 PE=1 SV=3 | 0.5395 | 0.912 | 0.8166 |
| 219 | sp\|P18418\|CALR_RAT | Calreticulin OS=Rattus norvegicus GN=Calr PE=1 SV=1 | 1.4723 | 1.2589 | 1.3932 |
| 220 | sp\|Q6AYC4\|CAPG_RAT | Macrophage-capping protein OS=Rattus norvegicus GN=Capg PE=1 SV=1 | 0.6982 | 0.8166 | 0.6668 |
| 221 | sp\|O35509\|RB11B_RAT | Ras-related protein Rab-11B OS=Rattus norvegicus GN=Rab11b PE=2 SV=4 | 1.4859 | 1.4322 | 1.1588 |
| 222 | sp\|Q62930\|CO9_RAT | Complement component C9 OS=Rattus norvegicus GN=C9 PE=2 SV=1 | 2.884 | 3.3419 | 2.7542 |
| 223 | sp\|P04639\|APOA1_RAT | Apolipoprotein A-I OS=Rattus norvegicus GN=Apoa1 PE=1 SV=2 | 2.0512 | 1.9409 | 1.556 |
| 224 | sp\|P54290\|CA2D1_RAT | Voltage-dependent calcium channel subunit alpha-2/delta-1 OS=Rattus norvegicus GN=Cacna2d1 PE=1 SV=1 | 0.6792 | 0.4831 | 0.7586 |
| 225 | sp\|B5DEH2\|ERLN2_RAT | Erlin-2 OS=Rattus norvegicus GN=Erlin2 PE=1 SV=1 | 1.406 | 1.1066 | 1.1803 |
| 226 | sp\|P47727\|CBR1_RAT | Carbonyl reductase [NADPH] 1 OS=Rattus norvegicus GN=Cbr1 PE=1 SV=2 | 2.1086 | 1.9055 | 2.208 |
| 227 | sp\|O35413\|SRBS2_RAT | Sorbin and SH3 domain-containing protein 2 OS=Rattus norvegicus GN=Sorbs2 PE=1 SV=2 | 0.6982 | 0.7379 | 0.879 |
| 228 | sp\|P38652\|PGM1_RAT | Phosphoglucomutase-1 OS=Rattus norvegicus GN=Pgm1 PE=1 SV=2 | 0.6194 | 1.1066 | 1.3062 |
| 229 | sp\|P42930\|HSPB1_RAT | Heat shock protein beta-1 OS=Rattus norvegicus GN=Hspb1 PE=1 SV=1 | 0.3873 | 0.8166 | 0.929 |
| 230 | sp\|Q5I0D1\|GLOD4_RAT | Glyoxalase domain-containing protein 4 OS=Rattus norvegicus GN=Glod4 PE=1 SV=1 | 0.6486 | 0.9204 | 0.8872 |
| 231 | sp\|Q66HA8\|HS105_RAT | Heat shock protein 105 kDa OS=Rattus norvegicus GN=Hsph1 PE=1 SV=1 | 0.6081 | 0.863 | 0.7178 |
| 232 | sp\|P50503\|F10A1_RAT | Hsc70-interacting protein OS=Rattus norvegicus GN=St13 PE=1 SV=1 | 0.5754 | 0.7798 | 0.955 |
| 233 | sp\|P27605\|HPRT_RAT | Hypoxanthine-guanine phosphoribosyltransferase OS=Rattus norvegicus GN=Hprt1 PE=1 SV=1 | 0.5702 | 0.871 | 0.9376 |
| 234 | sp\|P55053\|FABP5_RAT | Fatty acid-binding protein, epidermal OS=Rattus norvegicus GN=Fabp5 PE=1 SV=3 | 1.6904 | 6.4863 | 4.8306 |
| 235 | sp\|P25113\|PGAM1_RAT | Phosphoglycerate mutase 1 OS=Rattus norvegicus GN=Pgam1 PE=1 SV=4 | 0.5546 | 0.9204 | 0.9376 |
| 236 | sp\|P08289\|PPBT_RAT | Alkaline phosphatase, tissue-nonspecific isozyme OS=Rattus norvegicus GN=Alpl PE=1 SV=2 | 2.1281 | 2.1281 | 1.6444 |
| 237 | sp\|P55260\|ANXA4_RAT | Annexin A4 OS=Rattus norvegicus GN=Anxa4 PE=1 SV=3 | 1.8707 | 1.6749 | 1.4322 |
| 238 | sp\|Q8VHV7\|HNRH1_RAT | Heterogeneous nuclear ribonucleoprotein H OS=Rattus norvegicus GN=Hnrnph1 PE=1 SV=2 | 0.5598 | 0.9817 | 0.7943 |
| 239 | sp\|P31044\|PEBP1_RAT | Phosphatidylethanolamine-binding protein 1 OS=Rattus norvegicus GN=Pebp1 PE=1 SV=3 | 0.631 | 0.6026 | 0.6368 |
| 240 | sp\|Q9JLZ1\|GLRX3_RAT | Glutaredoxin-3 OS=Rattus norvegicus GN=Glrx3 PE=1 SV=2 | 0.4529 | 0.4875 | 0.6982 |
| 241 | sp\|P63102\|1433Z_RAT | 14-3-3 protein zeta/delta OS=Rattus norvegicus GN=Ywhaz PE=1 SV=1 | 0.3698 | 1.0666 | 0.8551 |
| 242 | sp\|P70645\|BLMH_RAT | Bleomycin hydrolase OS=Rattus norvegicus GN=Blmh PE=1 SV=1 | 0.6194 | 3.0761 | 3.1046 |
| 243 | sp\|P23562\|B3AT_RAT | Band 3 anion transport protein OS=Rattus norvegicus GN=Slc4a1 PE=2 SV=3 | 2.355 | 3.5318 | 1.9953 |
| 244 | sp\|Q00657\|CSPG4_RAT | Chondroitin sulfate proteoglycan 4 OS=Rattus norvegicus GN=Cspg4 PE=1 SV=2 | 0.6792 | 1.3062 | 1.0666 |
| 245 | sp\|P04256\|ROA1_RAT | Heterogeneous nuclear ribonucleoprotein A1 OS=Rattus norvegicus GN=Hnrnpa1 PE=1 SV=3 | 0.5916 | 1.0666 | 0.879 |
| 246 | sp\|P09811\|PYGL_RAT | Glycogen phosphorylase, liver form OS=Rattus norvegicus GN=Pygl PE=1 SV=5 | 0.6252 | 0.787 | 0.5754 |
| 247 | sp\|P27139\|CAH2_RAT | Carbonic anhydrase 2 OS=Rattus norvegicus GN=Ca2 PE=1 SV=2 | 2.0701 | 5.0119 | 4.0926 |
| 248 | sp\|P62703\|RS4X_RAT | 40S ribosomal protein S4, X isoform OS=Rattus norvegicus GN=Rps4x PE=2 SV=2 | 0.5105 | 0.4446 | 0.9817 |
| 249 | sp\|P97584\|PTGR1_RAT | Prostaglandin reductase 1 OS=Rattus norvegicus GN=Ptgr1 PE=2 SV=3 | 1.7701 | 2.6546 | 2.9648 |
| 250 | sp\|O88767\|PARK7_RAT | Protein DJ-1 OS=Rattus norvegicus GN=Park7 PE=1 SV=1 | 0.5105 | 1.1482 | 1.0093 |
| 251 | sp\|P19804\|NDKB_RAT | Nucleoside diphosphate kinase B OS=Rattus norvegicus GN=Nme2 PE=1 SV=1 | 0.6918 | 0.9908 | 1.1066 |
| 252 | sp\|Q6IRK9\|CBPQ_RAT | Carboxypeptidase Q OS=Rattus norvegicus GN=Cpq PE=1 SV=1 | 1.8535 | 0.8395 | 1.0471 |
| 253 | sp\|P20762\|IGG2C_RAT | Ig gamma-2C chain C region OS=Rattus norvegicus PE=2 SV=1 | 8.3176 | 8.9536 | 7.4473 |
| 254 | sp\|P29524\|PAI2_RAT | Plasminogen activator inhibitor 2 type A OS=Rattus norvegicus GN=Serpinb2 PE=2 SV=1 | 0.6546 | 0.787 | 1.3428 |
| 255 | sp\|Q6RUV5\|RAC1_RAT | Ras-related C3 botulinum toxin substrate 1 OS=Rattus norvegicus GN=Rac1 PE=1 SV=1 | 0.4571 | 0.9638 | 1.0765 |
| 256 | sp\|P60123\|RUVB1_RAT | RuvB-like 1 OS=Rattus norvegicus GN=Ruvbl1 PE=1 SV=1 | 1.7701 | 1.3804 | 0.9817 |
| 257 | sp\|Q9ERB4\|CSPG2_RAT | Versican core protein (Fragments) OS=Rattus norvegicus GN=Vcan PE=2 SV=2 | 1.4723 | 0.597 | 2.4434 |
| 258 | sp\|P51673\|RABP2_RAT | Cellular retinoic acid-binding protein 2 OS=Rattus norvegicus GN=Crabp2 PE=2 SV=2 | 0.6982 | 0.7586 | 1.0375 |
| 259 | sp\|P61589\|RHOA_RAT | Transforming protein RhoA OS=Rattus norvegicus GN=Rhoa PE=1 SV=1 | 0.5649 | 0.8395 | 0.8318 |
| 260 | sp\|P05426\|RL7_RAT | 60S ribosomal protein L7 OS=Rattus norvegicus GN=Rpl7 PE=1 SV=2 | 1.7378 | 0.5058 | 1.4588 |
| 261 | sp\|Q9R063\|PRDX5_RAT | Peroxiredoxin-5, mitochondrial OS=Rattus norvegicus GN=Prdx5 PE=1 SV=1 | 0.5649 | 0.7447 | 0.879 |
| 262 | sp\|P21396\|AOFA_RAT | Amine oxidase [flavin-containing] A OS=Rattus norvegicus GN=Maoa PE=1 SV=1 | 1.3428 | 0.7112 | 0.8954 |
| 263 | sp\|P05982\|NQO1_RAT | NAD(P)H dehydrogenase [quinone] 1 OS=Rattus norvegicus GN=Nqo1 PE=1 SV=4 | 2.0137 | 3.1333 | 2.8314 |
| 264 | sp\|P01835\|KACB_RAT | Ig kappa chain C region, B allele OS=Rattus norvegicus PE=1 SV=1 | 6.2517 | 7.7268 | 5.445 |
| 265 | sp\|Q5XI73\|GDIR1_RAT | Rho GDP-dissociation inhibitor 1 OS=Rattus norvegicus GN=Arhgdia PE=1 SV=1 | 0.6855 | 0.8954 | 0.871 |
| 266 | sp\|P12711\|ADHX_RAT | Alcohol dehydrogenase class-3 OS=Rattus norvegicus GN=Adh5 PE=1 SV=2 | 0.5702 | 0.787 | 0.9036 |
| 267 | sp\|P20761\|IGG2B_RAT | Ig gamma-2B chain C region OS=Rattus norvegicus GN=Igh-1a PE=1 SV=1 | 2.7797 | 3.281 | 2.6792 |
| 268 | sp\|Q9WVH8\|FBLN5_RAT | Fibulin-5 OS=Rattus norvegicus GN=Fbln5 PE=2 SV=1 | 1.4359 | 0.4169 | 1.2246 |
| 269 | sp\|Q920A6\|RISC_RAT | Retinoid-inducible serine carboxypeptidase OS=Rattus norvegicus GN=Scpep1 PE=2 SV=1 | 1.4859 | 1.3552 | 1.6293 |
| 270 | sp\|P45479\|PPT1_RAT | Palmitoyl-protein thioesterase 1 OS=Rattus norvegicus GN=Ppt1 PE=1 SV=1 | 1.6904 | 1.028 | 1.2474 |
| 271 | sp\|P09895\|RL5_RAT | 60S ribosomal protein L5 OS=Rattus norvegicus GN=Rpl5 PE=1 SV=3 | 1.406 | 1.0765 | 1.1169 |
| 272 | sp\|P11883\|AL3A1_RAT | Aldehyde dehydrogenase, dimeric NADP-preferring OS=Rattus norvegicus GN=Aldh3a1 PE=1 SV=3 | 0.2148 | 2.1677 | 1.6749 |
| 273 | sp\|P07895\|SODM_RAT | Superoxide dismutase [Mn], mitochondrial OS=Rattus norvegicus GN=Sod2 PE=1 SV=2 | 1.888 | 1.9409 | 1.6749 |
| 274 | sp\|P02651\|APOA4_RAT | Apolipoprotein A-IV OS=Rattus norvegicus GN=Apoa4 PE=2 SV=2 | 1.3062 | 1.9231 | 1.5704 |
| 275 | sp\|Q2LAP6\|TES_RAT | Testin OS=Rattus norvegicus GN=Tes PE=1 SV=1 | 0.1706 | 0.4365 | 0.5754 |
| 276 | sp\|A2VD12\|PBIP1_RAT | Pre-B-cell leukemia transcription factor-interacting protein 1 OS=Rattus norvegicus GN=Pbxip1 PE=2 SV=1 | 0.5808 | 0.3981 | 0.4875 |
| 277 | sp\|P26644\|APOH_RAT | Beta-2-glycoprotein 1 OS=Rattus norvegicus GN=Apoh PE=2 SV=2 | 2.466 | 2.6062 | 1.888 |
| 278 | sp\|P97590\|LEG7_RAT | Galectin-7 OS=Rattus norvegicus GN=Lgals7 PE=2 SV=4 | 0.6427 | 0.3981 | 0.5105 |
| 279 | sp\|P63331\|PP2AA_RAT | Serine/threonine-protein phosphatase 2A catalytic subunit alpha isoform OS=Rattus norvegicus GN=Ppp2ca PE=1 SV=1 | 0.6546 | 1.2589 | 1.1588 |
| 280 | sp\|Q920P6\|ADA_RAT | Adenosine deaminase OS=Rattus norvegicus GN=Ada PE=1 SV=3 | 3.7325 | 4.4875 | 3.3419 |
| 281 | sp\|Q6IFU8\|K1C17_RAT | Keratin, type I cytoskeletal 17 OS=Rattus norvegicus GN=Krt17 PE=2 SV=1 | 1.5276 | 0.597 | 0.955 |
| 282 | sp\|P62161\|CALM_RAT | Calmodulin OS=Rattus norvegicus GN=Calm1 PE=1 SV=2 | 0.5598 | 0.6918 | 0.6668 |
| 283 | sp\|Q9HB97\|PARVA_RAT | Alpha-parvin OS=Rattus norvegicus GN=Parva PE=1 SV=2 | 0.597 | 0.6081 | 0.8017 |
| 284 | sp\|P15865\|H14_RAT | Myosin phosphatase Rho-interacting protein OS=Rattus norvegicus GN=Mprip PE=1 SV=1 | 1.8197 | 0.0794 | 1.2474 |
| 285 | sp\|Q99068\|AMRP_RAT | Alpha-2-macroglobulin receptor-associated protein OS=Rattus norvegicus GN=Lrpap1 PE=1 SV=2 | 1.5417 | 0.8954 | 1.1912 |
| 286 | sp\|Q6PCU2\|VATE1_RAT | V-type proton ATPase subunit E 1 OS=Rattus norvegicus GN=Atp6v1e1 PE=1 SV=1 | 1.9588 | 1.5704 | 2.5586 |
| 287 | sp\|Q6DGG0\|PPID_RAT | Peptidyl-prolyl cis-trans isomerase D OS=Rattus norvegicus GN=Ppid PE=1 SV=3 | 0.3076 | 1.0471 | 0.6546 |
| 288 | sp\|P11232\|THIO_RAT | Thioredoxin OS=Rattus norvegicus GN=Txn PE=1 SV=2 | 0.3767 | 0.787 | 0.673 |
| 289 | sp\|P70623\|FABP4_RAT | Fatty acid-binding protein, adipocyte OS=Rattus norvegicus GN=Fabp4 PE=1 SV=3 | 3.3113 | 1.1588 | 2.355 |
| 290 | sp\|P62243\|RS8_RAT | 40S ribosomal protein S8 OS=Rattus norvegicus GN=Rps8 PE=1 SV=2 | 0.6546 | 0.4571 | 1.1482 |
| 291 | sp\|P48998\|INVO_RAT | Involucrin OS=Rattus norvegicus GN=Ivl PE=2 SV=1 | 2.3335 | 1.5996 | 1.5136 |
| 292 | sp\|O08619\|F13A_RAT | Coagulation factor XIII A chain OS=Rattus norvegicus GN=F13a1 PE=2 SV=3 | 2.9648 | 1.1482 | 1.5704 |
| 293 | sp\|B3DMA2\|ACD11_RAT | Acyl-CoA dehydrogenase family member 11 OS=Rattus norvegicus GN=Acad11 PE=1 SV=1 | 2.2491 | 1.9055 | 1.6904 |
| 294 | sp\|Q9QX79\|FETUB_RAT | Fetuin-B OS=Rattus norvegicus GN=Fetub PE=2 SV=1 | 1.3677 | 1.4191 | 1.2134 |
| 295 | sp\|Q29RW1\|MYH4_RAT | Myosin-4 OS=Rattus norvegicus GN=Myh4 PE=2 SV=1 | 3.6644 | 1.1169 | 4.0926 |
| 296 | sp\|P84092\|AP2M1_RAT | AP-2 complex subunit mu OS=Rattus norvegicus GN=Ap2m1 PE=1 SV=1 | 1.3062 | 0.673 | 1.0965 |
| 297 | sp\|Q8K3K4\|SPB10_RAT | Serpin B10 OS=Rattus norvegicus GN=Serpinb10 PE=2 SV=1 | 0.6918 | 2.3988 | 1.6144 |
| 298 | sp\|Q63377\|AT1B3_RAT | Sodium/potassium-transporting ATPase subunit beta-3 OS=Rattus norvegicus GN=Atp1b3 PE=2 SV=1 | 1.6749 | 1.9588 | 1.406 |
| 299 | sp\|Q6B345\|S10AB_RAT | Protein S100-A11 OS=Rattus norvegicus GN=S100a11 PE=2 SV=1 | 0.5012 | 1.3183 | 1.2942 |
| 300 | sp\|P10959\|EST1C_RAT | Carboxylesterase 1C OS=Rattus norvegicus GN=Ces1c PE=1 SV=3 | 1.4723 | 1.1066 | 0.7798 |
| 301 | sp\|Q6P7S1\|ASAH1_RAT | Acid ceramidase OS=Rattus norvegicus GN=Asah1 PE=2 SV=1 | 2.4889 | 1.8707 | 1.977 |
| 302 | sp\|Q6P7Q4\|LGUL_RAT | Lactoylglutathione lyase OS=Rattus norvegicus GN=Glo1 PE=1 SV=3 | 0.5058 | 1.0666 | 1.0471 |
| 303 | sp\|Q5M827\|PIR_RAT | Pirin OS=Rattus norvegicus GN=Pir PE=1 SV=1 | 0.5649 | 1.5849 | 1.2474 |
| 304 | sp\|P02650\|APOE_RAT | Apolipoprotein E OS=Rattus norvegicus GN=Apoe PE=1 SV=2 | 2.2284 | 2.0893 | 1.7219 |
| 305 | sp\|Q62867\|GGH_RAT | Histone H4 OS=Rattus norvegicus GN=Hist1h4b PE=1 SV=2 | 0.6026 | 0.879 | 2.0893 |
| 306 | sp\|Q9EQX9\|UBE2N_RAT | Ubiquitin-conjugating enzyme E2 N OS=Rattus norvegicus GN=Ube2n PE=1 SV=1 | 0.5861 | 0.871 | 1.0666 |
| 307 | sp\|P11167\|GTR1_RAT | Solute carrier family 2, facilitated glucose transporter member 1 OS=Rattus norvegicus GN=Slc2a1 PE=1 SV=1 | 1.5136 | 2.8576 | 1.8365 |
| 308 | sp\|P97687\|ENTP1_RAT | Ectonucleoside triphosphate diphosphohydrolase 1 OS=Rattus norvegicus GN=Entpd1 PE=1 SV=1 | 1.803 | 1.2589 | 0.9908 |
| 309 | sp\|P50116\|S10A9_RAT | Protein S100-A9 OS=Rattus norvegicus GN=S100a9 PE=1 SV=3 | 1.7219 | 3.8371 | 2.0324 |
| 310 | sp\|P06757\|ADH1_RAT | Alcohol dehydrogenase 1 OS=Rattus norvegicus GN=Adh1 PE=1 SV=3 | 0.6194 | 0.6607 | 1.2589 |
| 311 | sp\|Q6GMN2\|BAIP2_RAT | Brain-specific angiogenesis inhibitor 1-associated protein 2 OS=Rattus norvegicus GN=Baiap2 PE=1 SV=1 | 0.5346 | 0.7112 | 0.5546 |
| 312 | sp\|P30835\|K6PL_RAT | 6-phosphofructokinase, liver type OS=Rattus norvegicus GN=Pfkl PE=2 SV=3 | 0.5297 | 0.8091 | 0.6138 |
| 313 | sp\|P80067\|CATC_RAT | Dipeptidyl peptidase 1 OS=Rattus norvegicus GN=Ctsc PE=1 SV=3 | 1.7539 | 1.4191 | 1.3552 |
| 314 | sp\|P36972\|APT_RAT | Adenine phosphoribosyltransferase OS=Rattus norvegicus GN=Aprt PE=1 SV=1 | 0.4656 | 0.8091 | 1 |
| 315 | sp\|P97675\|ENPP3_RAT | Ectonucleotide pyrophosphatase/phosphodiesterase family member 3 OS=Rattus norvegicus GN=Enpp3 PE=1 SV=2 | 2.0701 | 1.803 | 1.6144 |
| 316 | sp\|P16573\|CEAM1_RAT | Carcinoembryonic antigen-related cell adhesion molecule 1 OS=Rattus norvegicus GN=Ceacam1 PE=1 SV=3 | 0.5754 | 1.028 | 1.0186 |
| 317 | sp\|Q5XIU9\|PGRC2_RAT | Membrane-associated progesterone receptor component 2 OS=Rattus norvegicus GN=Pgrmc2 PE=1 SV=1 | 2.2699 | 1.3804 | 1.1695 |
| 318 | sp\|Q64232\|TECR_RAT | Trans-2,3-enoyl-CoA reductase OS=Rattus norvegicus GN=Tecr PE=2 SV=1 | 1.7378 | 1.0471 | 1.4191 |
| 319 | sp\|Q9Z1H9\|PRDBP_RAT | Protein kinase C delta-binding protein OS=Rattus norvegicus GN=Prkcdbp PE=1 SV=1 | 1.6596 | 1.3804 | 1.3932 |
| 320 | sp\|P23764\|GPX3_RAT | Glutathione peroxidase 3 OS=Rattus norvegicus GN=Gpx3 PE=2 SV=2 | 1.8365 | 1.0765 | 1.2589 |
| 321 | sp\|P14659\|HSP72_RAT | Heat shock-related 70 kDa protein 2 OS=Rattus norvegicus GN=Hspa2 PE=2 SV=2 | 0.4246 | 0.6252 | 0.6982 |
| 322 | sp\|P00502\|GSTA1_RAT | Glutathione S-transferase alpha-1 OS=Rattus norvegicus GN=Gsta1 PE=1 SV=3 | 2.1878 | 2.0512 | 3.1915 |
| 323 | sp\|Q8CFN2\|CDC42_RAT | Cell division control protein 42 homolog OS=Rattus norvegicus GN=Cdc42 PE=1 SV=2 | 0.6427 | 1.4322 | 1.3428 |
| 324 | sp\|P15684\|AMPN_RAT | Aminopeptidase N OS=Rattus norvegicus GN=Anpep PE=1 SV=2 | 2.2909 | 1.888 | 1.888 |
| 325 | sp\|P30904\|MIF_RAT | Macrophage migration inhibitory factor OS=Rattus norvegicus GN=Mif PE=1 SV=4 | 0.5105 | 0.8241 | 0.9036 |
| 326 | sp\|P05369\|FPPS_RAT | Farnesyl pyrophosphate synthase OS=Rattus norvegicus GN=Fdps PE=2 SV=2 | 0.3733 | 0.8551 | 0.7112 |
| 327 | sp\|Q66H86\|OLFL1_RAT | Olfactomedin-like protein 1 OS=Rattus norvegicus GN=Olfml1 PE=2 SV=1 | 1.7701 | 0.5152 | 0.9376 |
| 328 | sp\|P05943\|S10AA_RAT | Protein S100-A10 OS=Rattus norvegicus GN=S100a10 PE=1 SV=2 | 1.8197 | 0.9908 | 1.3677 |
| 329 | sp\|P01041\|CYTB_RAT | Cystatin-B OS=Rattus norvegicus GN=Cstb PE=1 SV=1 | 0.6668 | 1.2474 | 1.1695 |
| 330 | sp\|Q4FZT6\|H2A3_RAT | Histone H2A type 3 OS=Rattus norvegicus PE=2 SV=3 | 0.413 | 1.3932 | 1.5136 |
| 331 | sp\|Q64122\|MYL9_RAT | Myosin regulatory light polypeptide 9 OS=Rattus norvegicus GN=Myl9 PE=1 SV=2 | 0.4966 | 1.4191 | 0.8551 |
| 332 | sp\|O35115\|FHL2_RAT | Four and a half LIM domains protein 2 OS=Rattus norvegicus GN=Fhl2 PE=1 SV=1 | 0.5445 | 2.1677 | 2.0512 |
| 333 | sp\|P29419\|ATP5I_RAT | ATP synthase subunit e, mitochondrial OS=Rattus norvegicus GN=Atp5i PE=1 SV=3 | 0.6138 | 0.5649 | 0.6607 |
| 334 | sp\|P20767\|LAC2_RAT | Ig lambda-2 chain C region OS=Rattus norvegicus PE=4 SV=1 | 1.7539 | 3.3113 | 2.3988 |
| 335 | sp\|P80254\|DOPD_RAT | D-dopachrome decarboxylase OS=Rattus norvegicus GN=Ddt PE=1 SV=3 | 0.3733 | 0.673 | 0.8872 |
| 336 | sp\|P11517\|HBB2_RAT | Hemoglobin subunit beta-2 OS=Rattus norvegicus PE=1 SV=2 | 3.0479 | 4.8306 | 2.5119 |
| 337 | sp\|Q561R0\|PERP1_RAT | Plasma cell-induced resident endoplasmic reticulum protein OS=Rattus norvegicus GN=Pacap PE=2 SV=1 | 2.4889 | 0.4613 | 0.7112 |
| 338 | sp\|P04644\|RS17_RAT | 40S ribosomal protein S17 OS=Rattus norvegicus GN=Rps17 PE=2 SV=3 | 0.6668 | 0.9817 | 1.0666 |
| 339 | sp\|P29826\|HB2B_RAT | Rano class II histocompatibility antigen, B-1 beta chain OS=Rattus norvegicus GN=RT1-Bb PE=2 SV=1 | 0.5754 | 1.1695 | 1.1803 |
| 340 | sp\|B0BNE5\|ESTD_RAT | S-formylglutathione hydrolase OS=Rattus norvegicus GN=Esd PE=1 SV=1 | 0.4875 | 0.6982 | 0.6026 |
| 341 | sp\|Q811A3\|PLOD2_RAT | Procollagen-lysine,2-oxoglutarate 5-dioxygenase 2 OS=Rattus norvegicus GN=Plod2 PE=2 SV=1 | 1.7061 | 1.8535 | 1.9953 |
| 342 | sp\|P63029\|TCTP_RAT | Translationally-controlled tumor protein OS=Rattus norvegicus GN=Tpt1 PE=1 SV=1 | 0.4446 | 0.955 | 1.1169 |
| 343 | sp\|P11030\|ACBP_RAT | Acyl-CoA-binding protein OS=Rattus norvegicus GN=Dbi PE=1 SV=3 | 0.5012 | 0.7798 | 0.7516 |
| 344 | sp\|P04961\|PCNA_RAT | Proliferating cell nuclear antigen OS=Rattus norvegicus GN=Pcna PE=1 SV=1 | 0.6898 | 1.0765 | 1.1066 |
| 345 | sp\|Q9QYL8\|LYPA2_RAT | Acyl-protein thioesterase 2 OS=Rattus norvegicus GN=Lypla2 PE=1 SV=1 | 0.5754 | 0.5808 | 0.5346 |
| 346 | sp\|Q80XX4\|MSTN1_RAT | Musculoskeletal embryonic nuclear protein 1 OS=Rattus norvegicus GN=Mustn1 PE=2 SV=1 | 0.4018 | 0.5152 | 0.5012 |
| 347 | sp\|Q63ZV7\|SWI5_RAT | DNA repair protein SWI5 homolog OS=Rattus norvegicus GN=Swi5 PE=2 SV=1 | 0.3873 | 0.6194 | 0.7798 |
| 348 | sp\|Q9WUW3\|CFAI_RAT | Complement factor I OS=Rattus norvegicus GN=Cfi PE=2 SV=1 | 1.6904 | 2.2909 | 2.6062 |
| 349 | sp\|Q10743\|ADA10_RAT | Disintegrin and metalloproteinase domain-containing protein 10 (Fragment) OS=Rattus norvegicus GN=Adam10 PE=2 SV=1 | 1.9231 | 1.1912 | 1.1695 |
| 350 | sp\|P12749\|RL26_RAT | 60S ribosomal protein L26 OS=Rattus norvegicus GN=Rpl26 PE=1 SV=1 | 0.597 | 0.5248 | 1.3428 |
| 351 | sp\|P36953\|AFAM_RAT | Afamin OS=Rattus norvegicus GN=Afm PE=2 SV=1 | 0.5546 | 0.4571 | 1.028 |
| 352 | sp\|P04638\|APOA2_RAT | Apolipoprotein A-II OS=Rattus norvegicus GN=Apoa2 PE=2 SV=1 | 1.5849 | 2.2699 | 1.4997 |
| 353 | sp\|P58775\|TPM2_RAT | Tropomyosin beta chain OS=Rattus norvegicus GN=Tpm2 PE=2 SV=1 | 0.52 | 0.5808 | 0.7447 |
| 354 | sp\|Q5FWT5\|GATA_RAT | Glutamyl-tRNA(Gln) amidotransferase subunit A, mitochondrial OS=Rattus norvegicus GN=Qrsl1 PE=2 SV=1 | 0.6081 | 0.7516 | 0.8241 |
| 355 | sp\|Q7TP52\|CMBL_RAT | Carboxymethylenebutenolidase homolog OS=Rattus norvegicus GN=Cmbl PE=2 SV=1 | 1.7865 | 1.4191 | 1.1482 |
| 356 | sp\|P06907\|MYP0_RAT | Myelin protein P0 OS=Rattus norvegicus GN=Mpz PE=1 SV=1 | 1.977 | 1.0666 | 1.1803 |
| 357 | sp\|Q62639\|RHEB_RAT | GTP-binding protein Rheb OS=Rattus norvegicus GN=Rheb PE=1 SV=1 | 1.7378 | 1.1169 | 1.1695 |
| 358 | sp\|P02600\|MYL1_RAT | Myosin light chain 1/3, skeletal muscle isoform OS=Rattus norvegicus GN=Myl1 PE=1 SV=2 | 2.1478 | 1.1695 | 5.2481 |
| 359 | sp\|Q5BJP6\|RRF2M_RAT | Ribosome-releasing factor 2, mitochondrial OS=Rattus norvegicus GN=Gfm2 PE=2 SV=2 | 0.6194 | 0.9204 | 1.3062 |
| 360 | sp\|P47824\|P2RX1_RAT | P2X purinoceptor 1 OS=Rattus norvegicus GN=P2rx1 PE=2 SV=1 | 0.631 | 0.929 | 1.0186 |
| 361 | sp\|Q6UE39\|GLT13_RAT | Polypeptide N-acetylgalactosaminyltransferase 13 OS=Rattus norvegicus GN=Galnt13 PE=2 SV=1 | 0.6427 | 0.8472 | 0.787 |
| 362 | sp\|Q5PPJ4\|DOHH_RAT | Deoxyhypusine hydroxylase OS=Rattus norvegicus GN=Dohh PE=2 SV=1 | 0.6081 | 0.7516 | 0.5395 |
| 363 | sp\|Q925G0\|RBM3_RAT | Putative RNA-binding protein 3 OS=Rattus norvegicus GN=Rbm3 PE=1 SV=2 | 0.6982 | 0.6918 | 0.955 |
| 364 | sp\|Q9QZC5\|GRB7_RAT | Growth factor receptor-bound protein 7 OS=Rattus norvegicus GN=Grb7 PE=1 SV=1 | 0.6368 | 1.0965 | 0.8395 |
| 365 | sp\|Q8CGS4\|CHMP3_RAT | Charged multivesicular body protein 3 OS=Rattus norvegicus GN=Chmp3 PE=1 SV=3 | 0.6081 | 0.5808 | 0.7311 |
| 366 | sp\|Q499U2\|ELMO3_RAT | Engulfment and cell motility protein 3 OS=Rattus norvegicus GN=Elmo3 PE=2 SV=1 | 1.6293 | 1.3183 | 1.0471 |
| 367 | sp\|Q5BJQ2\|FA63A_RAT | Protein FAM63A OS=Rattus norvegicus GN=Fam63a PE=2 SV=1 | 0.631 | 0.8166 | 0.8166 |
| 368 | sp\|P31721\|C1QB_RAT | Complement C1q subcomponent subunit B OS=Rattus norvegicus GN=C1qb PE=1 SV=2 | 2.2284 | 2.0512 | 2.0512 |
| 369 | sp\|P63055\|PCP4_RAT | Purkinje cell protein 4 OS=Rattus norvegicus GN=Pcp4 PE=1 SV=2 | 0.4055 | 0.7727 | 0.7379 |
| 370 | sp\|P04550\|PTMS_RAT | Parathymosin OS=Rattus norvegicus GN=Ptms PE=1 SV=2 | 0.597 | 0.8017 | 0.7516 |
| 371 | sp\|Q5HZW7\|PLET1_RAT | Placenta-expressed transcript 1 protein OS=Rattus norvegicus GN=Plet1 PE=2 SV=1 | 1.4322 | 1.5849 | 1.406 |
| 372 | sp\|P20291\|AL5AP_RAT | Arachidonate 5-lipoxygenase-activating protein OS=Rattus norvegicus GN=Alox5ap PE=1 SV=2 | 2.2699 | 1.1803 | 1.4322 |
| 373 | sp\|P25409\|ALAT1_RAT | Alanine aminotransferase 1 OS=Rattus norvegicus GN=Gpt PE=1 SV=2 | 0.4446 | 0.7047 | 0.787 |
| 374 | sp\|P42260\|GRIK2_RAT | Glutamate receptor, ionotropic kainate 2 OS=Rattus norvegicus GN=Grik2 PE=1 SV=2 | 0.6668 | 0.7727 | 0.8872 |
| 375 | sp\|Q6P7C7\|GPNMB_RAT | Transmembrane glycoprotein NMB OS=Rattus norvegicus GN=Gpnmb PE=2 SV=1 | 4.8753 | 2.6546 | 1.4859 |
| 376 | sp\|Q8K4K5\|L2GL1_RAT | Lethal(2) giant larvae protein homolog 1 OS=Rattus norvegicus GN=Llgl1 PE=2 SV=1 | 0.5598 | 0.7244 | 0.6252 |
| 377 | sp\|Q62645\|NMDE4_RAT | Glutamate [NMDA] receptor subunit epsilon-4 OS=Rattus norvegicus GN=Grin2d PE=1 SV=2 | 1.8707 | 1.3677 | 1.1588 |
| 378 | sp\|Q66H84\|MAPK3_RAT | MAP kinase-activated protein kinase 3 OS=Rattus norvegicus GN=Mapkapk3 PE=2 SV=1 | 0.6792 | 1.2246 | 1.1066 |
| 379 | sp\|P30919\|ASPG_RAT | N(4)-(Beta-N-acetylglucosaminyl)-L-asparaginase OS=Rattus norvegicus GN=Aga PE=1 SV=2 | 1.4588 | 1.9409 | 1.1169 |
| 380 | sp\|Q6IFW2\|K1C40_RAT | Keratin, type I cytoskeletal 40 OS=Rattus norvegicus GN=Krt40 PE=2 SV=1 | 1.9231 | 0.6368 | 0.5495 |
| 381 | sp\|Q7TSE9\|HAX1_RAT | HCLS1-associated protein X-1 OS=Rattus norvegicus GN=Hax1 PE=1 SV=1 | 1.406 | 0.9908 | 1.1695 |
| 382 | sp\|P04903\|GSTA2_RAT | Glutathione S-transferase alpha-2 OS=Rattus norvegicus GN=Gsta2 PE=2 SV=2 | 2.5351 | 2.355 | 2.7797 |
| 383 | sp\|P25886\|RL29_RAT | 60S ribosomal protein L29 OS=Rattus norvegicus GN=Rpl29 PE=1 SV=3 | 1.9409 | 0.0597 | 1.3062 |
| 384 | sp\|Q64LC9\|RBM4B_RAT | RNA-binding protein 4B OS=Rattus norvegicus GN=Rbm4b PE=2 SV=2 | 1.9231 | 1.3428 | 1.4588 |
| 385 | sp\|Q925D4\|T176B_RAT | Transmembrane protein 176B OS=Rattus norvegicus GN=Tmem176b PE=1 SV=1 | 0.5012 | 0.6194 | 0.3631 |
| 386 | sp\|O88277\|FAT2_RAT | Protocadherin Fat 2 OS=Rattus norvegicus GN=Fat2 PE=1 SV=1 | 0.6081 | 0.7447 | 0.7656 |
| 387 | sp\|Q6PEC0\|AP4A_RAT | Bis(5'-nucleosyl)-tetraphosphatase [asymmetrical] OS=Rattus norvegicus GN=Nudt2 PE=2 SV=3 | 0.52 | 0.8472 | 0.9462 |
| 388 | sp\|Q9WTQ2\|PODXL_RAT | Podocalyxin OS=Rattus norvegicus GN=Podxl PE=1 SV=2 | 0.6855 | 0.6918 | 0.8551 |
| 389 | sp\|P06766\|DPOLB_RAT | DNA polymerase beta OS=Rattus norvegicus GN=Polb PE=1 SV=4 | 0.6138 | 0.8472 | 0.6252 |
| 390 | sp\|Q64566\|AT2C1_RAT | Calcium-transporting ATPase type 2C member 1 OS=Rattus norvegicus GN=Atp2c1 PE=2 SV=1 | 1.7865 | 1.6749 | 1.0375 |
| 391 | sp\|P23565\|AINX_RAT | Alpha-internexin OS=Rattus norvegicus GN=Ina PE=1 SV=2 | 2.4434 | 1.0568 | 1.1912 |
| 392 | sp\|Q9WUC8\|PLRG1_RAT | Pleiotropic regulator 1 OS=Rattus norvegicus GN=Plrg1 PE=2 SV=1 | 1.9588 | 1.7865 | 1.8197 |
| 393 | sp\|Q63633\|S12A5_RAT | Solute carrier family 12 member 5 OS=Rattus norvegicus GN=Slc12a5 PE=1 SV=2 | 0.6368 | 0.6194 | 0.6252 |
| 394 | RRRRRsp\|Q6MG62\|MSH5_RAT | REVERSED MutS protein homolog 5 OS=Rattus norvegicus GN=Msh5 PE=2 SV=1 | 1.9953 | 1.2823 | 1.3552 |
| 395 | sp\|Q562C7\|K0020_RAT | Pumilio domain-containing protein KIAA0020 homolog OS=Rattus norvegicus PE=2 SV=1 | 2.4434 | 1.0568 | 1.1912 |
| 396 | sp\|Q9ERA7\|MSLN_RAT | Mesothelin OS=Rattus norvegicus GN=Msln PE=2 SV=2 | 1.4322 | 1.028 | 1.0965 |
| 397 | sp\|P63035\|CYH2_RAT | Cytohesin-2 OS=Rattus norvegicus GN=Cyth2 PE=1 SV=1 | 2.1281 | 1.8707 | 1.3062 |
| 398 | RRRRRsp\|P27881\|HXK2_RAT | REVERSED Hexokinase-2 OS=Rattus norvegicus GN=Hk2 PE=1 SV=1 | 1.4588 | 0.9817 | 1.2942 |
| 399 | sp\|Q5XIC4\|SPG21_RAT | Maspardin OS=Rattus norvegicus GN=Spg21 PE=2 SV=1 | 1.7865 | 1.1169 | 1.4322 |
| 400 | sp\|Q5U2Z2\|AJUBA_RAT | LIM domain-containing protein ajuba OS=Rattus norvegicus GN=Ajuba PE=1 SV=1 | 1.406 | 0.9204 | 0.9376 |
| 401 | sp\|P27590\|UROM_RAT | Uromodulin OS=Rattus norvegicus GN=Umod PE=2 SV=1 | 0.6918 | 1.0186 | 0.7178 |
| 402 | sp\|Q6AYQ8\|FAHD1_RAT | Acylpyruvase FAHD1, mitochondrial OS=Rattus norvegicus GN=Fahd1 PE=2 SV=1 | 0.5702 | 0.9638 | 0.7047 |
